# Distinct cervical tissue-adherent and luminal microbiome communities correlate with mucosal host gene expression and protein levels in Kenyan sex workers

**DOI:** 10.1186/s40168-023-01502-4

**Published:** 2023-03-31

**Authors:** Gabriella Edfeldt, Vilde Kaldhusdal, Paulo Czarnewski, Frideborg Bradley, Sofia Bergström, Julie Lajoie, Jiawu Xu, Anna Månberg, Joshua Kimani, Julius Oyugi, Peter Nilsson, Annelie Tjernlund, Keith R. Fowke, Douglas S. Kwon, Kristina Broliden

**Affiliations:** 1 Department of Medicine Solna, Division of Infectious Diseases, Karolinska Institutet, Department of Infectious Diseases, Karolinska University Hospital, Center for Molecular Medicine, J7:20, S-171 76 Stockholm, Sweden; 2 Department of Biochemistry and Biophysics, National Bioinformatics Infrastructure Sweden, SciLifeLab, Stockholm University, Solna, Sweden; 3 Division of Affinity Proteomics, Department of Protein Science, SciLifeLab, KTH Royal Institute of Technology, Stockholm, Sweden; 4 Department of Medical Microbiology and Infectious Diseases, University of Manitoba, Winnipeg, Canada; 5 Ragon Institute of MGH, MIT and Harvard, Cambridge, MA USA; 6 Department of Medical Microbiology, University of Nairobi, Nairobi, Kenya; 7 Partners for Health and Development in Africa, Nairobi, Kenya; 8 Department of Community Health Sciences, University of Manitoba, Winnipeg, Canada

**Keywords:** Cervix, Ectocervix, Microbiota, 16S rRNA gene, Tissue, Tissue-adherent, Biofilm, Luminal, Transcriptomics, Protein profiling

## Abstract

**Background:**

The majority of studies characterizing female genital tract microbiota have focused on luminal organisms, while the presence and impact of tissue-adherent ectocervical microbiota remain incompletely understood. Studies of luminal and tissue-associated bacteria in the gastrointestinal tract suggest that these communities may have distinct roles in health and disease. Here, we performed a multi-omics characterization of paired luminal and tissue samples collected from a cohort of Kenyan female sex workers.

**Results:**

We identified a tissue-adherent bacterial microbiome, with a higher alpha diversity than the luminal microbiome, in which dominant genera overall included *Gardnerella* and *Lactobacillus*, followed by *Prevotella*, *Atopobium*, and *Sneathia*. About half of the *L. iners-*dominated luminal samples had a corresponding *Gardnerella-*dominated tissue microbiome. Broadly, the tissue-adherent microbiome was associated with fewer differentially expressed host genes than the luminal microbiome. Gene set enrichment analysis revealed that *L. crispatus-*dominated tissue-adherent communities were associated with protein translation and antimicrobial activity, whereas a highly diverse microbial community was associated with epithelial remodeling and pro-inflammatory pathways. Tissue-adherent communities dominated by *L. iners* and *Gardnerella* were associated with lower host transcriptional activity. Tissue-adherent microbiomes dominated by *Lactobacillus* and *Gardnerella* correlated with host protein profiles associated with epithelial barrier stability, although with a more pro-inflammatory profile for the *Gardnerella*-dominated microbiome group. Tissue samples with a highly diverse composition had a protein profile representing cell proliferation and pro-inflammatory activity.

**Conclusion:**

We identified ectocervical tissue-adherent bacterial communities in all study participants of a female sex worker cohort. These communities were distinct from cervicovaginal luminal microbiota in a significant proportion of individuals. We further revealed that bacterial communities at both sites correlated with distinct host gene expression and protein levels. The tissue-adherent bacterial community could possibly act as a reservoir that seed the lumen with less optimal, non-*Lactobacillus*, bacteria.

Video Abstract

**Supplementary Information:**

The online version contains supplementary material available at 10.1186/s40168-023-01502-4.

## Background

The cervicovaginal microbiome impacts a number of important reproductive outcomes, including preterm birth, cervicitis, fertility, and susceptibility to sexually transmitted infections including HIV [1–8]. The molecular mechanisms that underlie host-microbiota interactions at mucosal sites have informed the development of new preventative and therapeutic measures against many diseases. The composition of the mucosal microbiome is thus of high clinical relevance, which was also illustrated by a fourfold increase in the rate of HIV infection in African women with a highly diverse cervicovaginal microbiota compared to those with a *L. crispatus*-dominated community [9]. While a cervicovaginal microbiota dominated by *L. crispatus* is optimal, African women have high prevalence of diverse bacterial communities [10].

A *Lactobacillus*-dominated cervicovaginal microbiota maintains an acidic luminal environment that promotes antimicrobial immunity and epithelial barrier function. In contrast, polymicrobial genital communities with low *Lactobacillus* abundance are associated with increased levels of pro-inflammatory proteins, higher numbers of activated cervical CD4^+^ T cells, and disruption of the epithelial lining [9, 11–13]. Other host responses to microbiota include epigenetic regulation, autophagy, and stress in epithelial cells [14, 15]). Different host transcriptome and protein expression have recently been linked to treatment response in women with bacterial vaginosis (BV) [16]. Although tissue-adherent pathogens are usually quickly eradicated by a high epithelial cell turnover, they can secrete factors that overcome the host defenses. Biofilm formation on mucosal surfaces is one such defense strategy.

Tissue-associated microbiota in the intestine may differ significantly from their luminal counterparts both taxonomically and in terms of impact on the host [17]. There is little characterization of the ectocervical tissue-adherent microbiome, and even less on its relevance to host mucosal defenses in the female genital tract [18]. Although practical factors limit the sampling of genital tissue from women to investigate these factors, studies on the impact of the microbiome on the female genital mucosa are needed, especially for populations at high risk of sexually transmitted infections. Using a unique set of paired ectocervical tissue biopsies and cervicovaginal lavage samples from a clinical cohort of Kenyan female sex workers, we applied a multi-omics approach to identify the luminal and tissue-associated microbiome in the female genital tract and to characterize associations between microbiota and host function.

## Results

### Kenyan sex workers show a high prevalence of diverse microbial communities in cervicovaginal lavage samples

**Fig. 1:**
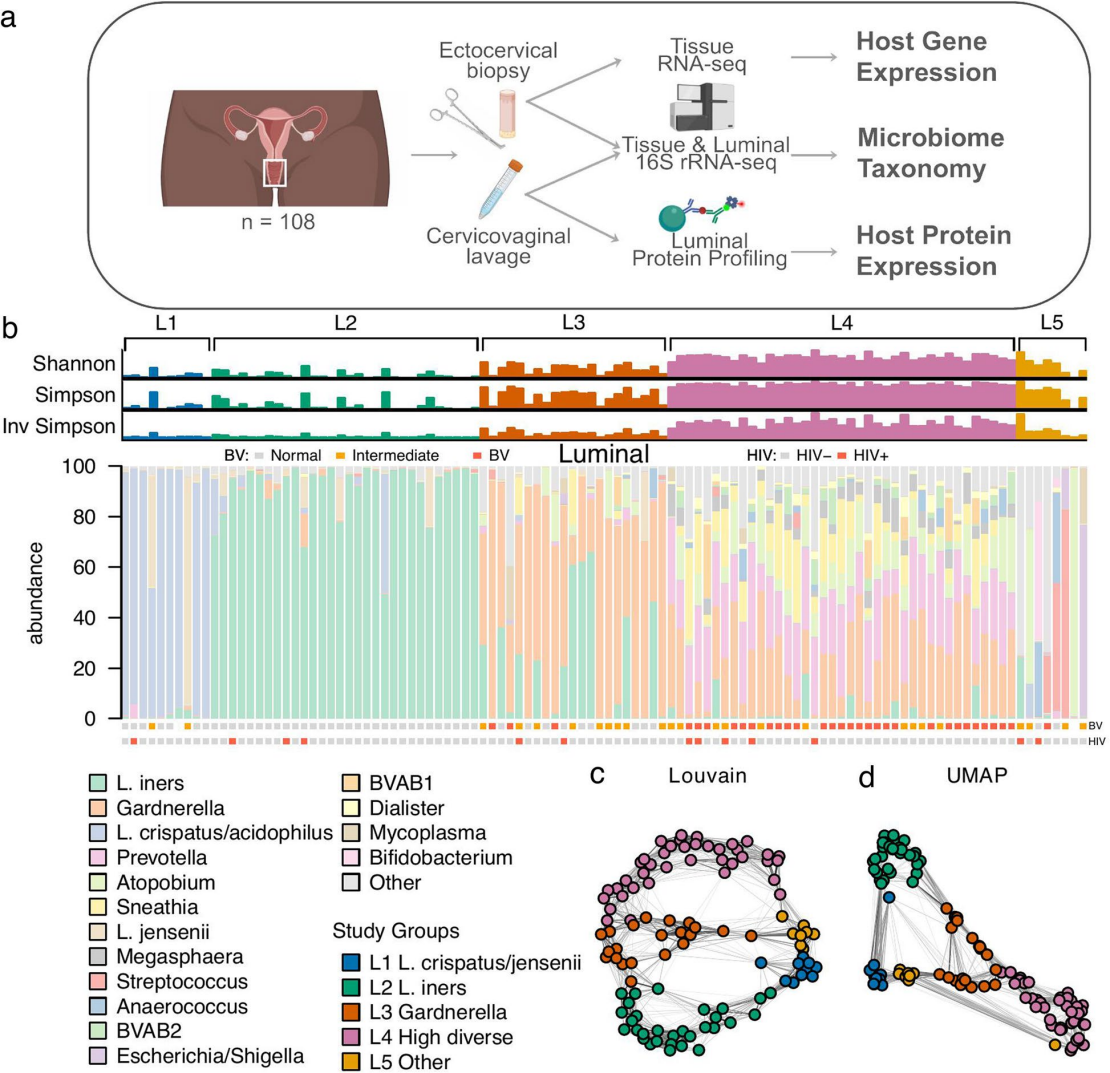
Characterization of a highly diverse microbiome in cervicovaginal (luminal) samples from Kenyan sex workers. Cervicovaginal lavage (luminal) and ectocervical tissue study samples were assessed by 16S rRNA sequencing, gene expression, and protein profiling. **a** Schematic drawing depicting the sampling scheme and the resulting omics datasets. **b** Bar plots of alpha diversity indices and taxonomy profiles for each individual luminal sample. Color-coded squares above the stacked bar plots indicate bacterial vaginosis (BV, binned Nugent’s scores): Gray: negative, orange: intermediate, red: positive; and HIV diagnosis: Gray: HIV seronegative, red: HIV seropositive. Two12-SNN graphs were constructed using: **c** Louvain community detection algorithm, and **d** Uniform Manifold Approximation (UMAP). The two graphs were overlayed in color with the predefined luminal study groups. The undirected edges are included in gray connecting the nodes

A total of 108 women from the Pumwani Sex Worker Cohort, Nairobi, Kenya, were enrolled into the present study. Criteria for enrollment were premenopausal women, 18–50 years of age, no prior hysterectomy, not pregnant or breastfeeding, and negative for infection with *Treponema pallidum*, *Trichomonas vaginalis, Neisseria gonorrhoeae*, and *Chlamydia trachomatis*. Paired samples of ectocervical tissue and cervicovaginal lavage (“luminal”) were collected 2 weeks after they began a monitored 4-week period of sexual abstinence. All mucosal samples were processed for 16S rRNA V4 gene sequencing; the ectocervical tissue samples were processed for transcriptional (mRNA) profiling and the luminal samples for protein profiling. This design represents a unique clinical cohort with a multi-omics-based characterization of matching tissue biopsies and cervical secretions to better understand host-microbe mucosal interactions (Fig. 1a).

Across all samples, we found a highly heterogeneous luminal microbiome. The most prevalent genus was *Lactobacillus* with a relative abundance of 41% (with *L. iners* representing 30% and *L. crispatus* 9%), followed by *Gardnerella* (22%), *Prevotella* (9%), *Atopobium* (6%), and *Sneathia* (5%) (Fig. 1b; Suppl. Table 1). The bacterial transcriptional activity is not linear to its relative abundance in a community [19, 20]; thus, even low abundant bacteria can exert an effect on its environment and host. For the following analyses, the luminal samples (designated “L”) were classified into five supervised groups. This classification was adapted from the community type classification previously published by us [9], aiming to clearly separate *Lactobacillus*, *Gardnerella* and highly diverse community; 10 samples with > 80% relative abundance of *L. crispatus* and/or *L. jensenii* were assigned to L1 (*L.* c*rispatus*); 30 samples with > 80% *Lactobacillus* spp. (mainly *L. iners)* were assigned to L2 (*L. iners*); 21 samples with > 10% *Gardnerella* and < 5% *Prevotella* were assigned to L3 (*Gardnerella*); 39 samples with >5% *Prevotella* were assigned to L4 (highly diverse); 8 samples did not reach the *Lactobacillus*, *Gardnerella* or *Prevotella* thresholds and were thus assigned L5 (other). The samples in L5 group were non-homogenous and presented with the following majority bacteria; *Atopobium* (99% and 61%), *Escherichia*/*Shigella* (76%), *Bifidobacterium* (55%), *L. iners* (24%), *L. gasseri* (50%), and *Streptococcus* (51% and 83%). In agreement with the observed bacterial abundance, the two *Lactobacillus*-dominated groups (L1 and L2) had lower alpha diversity, while groups L3 and L5 had intermediate diversity and group L4 had the highest diversity (Fig. 1b). Confirming the validity of our supervised group definitions, unsupervised clustering with Louvain and dimensionality reduction with UMAP supported the classification categories (Fig. 1c–d)*.* This grouping structure also correlated well with a diagnosis of BV; 72% of women clinically diagnosed with BV belonged to L4, and none of the BV cases belonged to L1 or L2 (Table 1).

**Table 1:** Sociodemographic and clinical characteristics of study participants at time of sample collection, grouped based on their luminal microbiome

	Study groups						
	L1	L2	L3	L4	L5	***p***-value^a^	Data n/a^b^
	(*n*=10)	(*n*=30)	(*n*=21)	(*n*=39)	(*n*=8)		
	Median (range or %)						Number
***Sociodemographic parameters***							
**Age** (years)	32 (23–47)	32 (21–48)	37 (20–48)	33 (21–50)	34 (24–40)	0.35^1^	0
**Time in sex work** (months)	30 (12–120)	30 (8–264)	102 (12–372)	36 (3–324)	30 (12–144)	0.25^1^	2
**Number of weekly clients** ^c^	9 (3–25)	4 (1–50)	5 (0–30)	4 (0–50)	7 (2–28)	0.2^1^	7
**Marital status** (married)	3 (30%)	4 (13%)	4 (19%)	10 (26%)	3 (38%)	0.55^2^	2
**Children** (number)	2 (0–4)	2 (1–4)	2 (1–4)	2 (0–4)	2 (1–5)	0.79^1^	15
**Educational level** (years in school)	9 (7–16)	10 (7–15)	9 (7–15)	10 (2–21)	10 (6–15)	0.94^1^	1
***Sex hormone status***							
**DMPA use** (yes)	3 (30%)	11 (37%)	7 (33%)	8 (21%)	5 (62%)	0.19^2^	0
**Progesterone** ^d^ (ng/mL)	0.05(0.05–0.05)	0.05(0.05–0.05)	0.05(0.05–0.09)	0.05(0.05–0.05)	0.05(0.05–0.05)	0.36^1^	2
**Estradiol** ^d^ (pg/mL)	22 (22–68)	22 (22–124)	29 (22–35)	22 (22–92)	22 (22–57)	0.97^1^	2
**Time since onset of menses** ^e^ (days)	16 (4–44)	8 (5–40)	9 (5–30)	9 (3–34)	14 (6–19)	0.29^1^	3
**Progesterone** ^f^ (ng/mL)	3(0.05–8)	0.05(0.05–9)	0.06(0.05–19)	0.05(0.05–11)	0.15(0.05–10)	0.09^1^	0
**Estradiol** ^f^ (pg/mL)	215 (36–296)	76 (22–258)	140 (22–405)	82 (22–290)	109 (22–248)	0.09^1^	0
***STIs and vaginal health***							
**HIV serostatus** (seropositive)	1 (10%)	3 (10%)	2 (10%)	5 (13%)	2 (25%)	0.81^2^	0
**Presence of NG** ^g^	0	0	0	0	0		0
**Presence of CT** ^g^	0	0	0	0	0		0
**Presence of yeast** ^g^	0	1 (3%)	0	1 (3%)	1 (12%)	0.37^2^	2
**Vaginal discharge** ^g^	3 (30%)	3 (10%)	1 (5%)	2 (5%)	0	0.1^2^	5
**Nugents’ score (bacterial vaginosis)** ^h^						<0.001^2^	2
-Negative (0–3)	7 (70%)	30 (100%)	9 (43%)	1 (3%)	2 (25%)		
*-*Intermediate (4–6)	2 (20%)	0	9 (43%)	10 (26%)	4 (50%)		
*-*Positive (7–10)	0	0	3 (14%)	28 (72%)	1 (12%)		
***Antibiotic use*** ^i^	1	3	4	5	3	0.35^2^	0

^a^ *p*-values: ^1^Kruskal-Wallis rank sum test; ^2^Fisher’s exact test

^b^Data n/a: Data not available for number of samples

^c^Number of weekly clients: Data from the questionnaire two weeks prior to sample collection: “How many clients did you have the past 7 days?”

^d^Plasma concentration of progesterone (P4, lower limit of detection=0.05 ng/mL) and estradiol (E2, lower limit of detection 22 pg/mL) in study participants using DMPA

^e^Time since onset of last menses (days) for study participants not using hormonal contraceptives (the control group)

^f^Plasma concentration of progesterone (P4, lower limit of detection=0.05 ng/mL) and estradiol (E2, lower limit of detection 22 pg/mL) in study participants not using hormonal contraceptives (the control group)

^g^Having an ongoing STI at time of enrolment was an exclusion criteria for participating in the study. None of the study participants were diagnosed with neither *C.trachomatis* nor *N. gonorroheae* at time of sample collection. The scoring for yeast was made on the Gram-stained slide used for BV. Presence of discharge was recorded during physical examination

^h^Bacterial vaginosis: the statistical analysis is based on BV diagnosis (Nugent’s score 7–10) (yes/no) ^i^Antibiotic use was defined from the medical records as prescribed at the last visit (2 weeks prior to sample collection), thus the numbers refer to ongoing or recently finalized antibiotic treatment. The HIV-seropositive participants were all on regular treatment with co-trimoxazole. The other three study participants received either amoxicillin or azithromycin

Interrogating clinical covariates (Suppl. Table 2) revealed that neither age, time engaged in sex work, number of weekly clients, marital status, number of children, nor educational level were significantly different across the L1–L5 groups (*p* > 0.05) (Table 1). For women using the hormonal contraceptive depot medroxyprogesterone acetate (DMPA), we saw no difference across the groups based either on DMPA usage per se or on progesterone or estradiol levels (*p* > 0.05). For women with natural menstrual cycles, time since the onset of last menses and progesterone or estradiol levels were also similar across the L1–L5 groups (*p* > 0.05). The use of antibiotics was limited and no differences between the study groups were observed (*p* > 0.05). Among parameters defining genital health, only BV differed significantly between the groups as expected (*p* < 0.001) (Table 1; Fig. 1b).

### Bacterial communities demonstrate mutual exclusivity across luminal samples

**Fig. 2:**
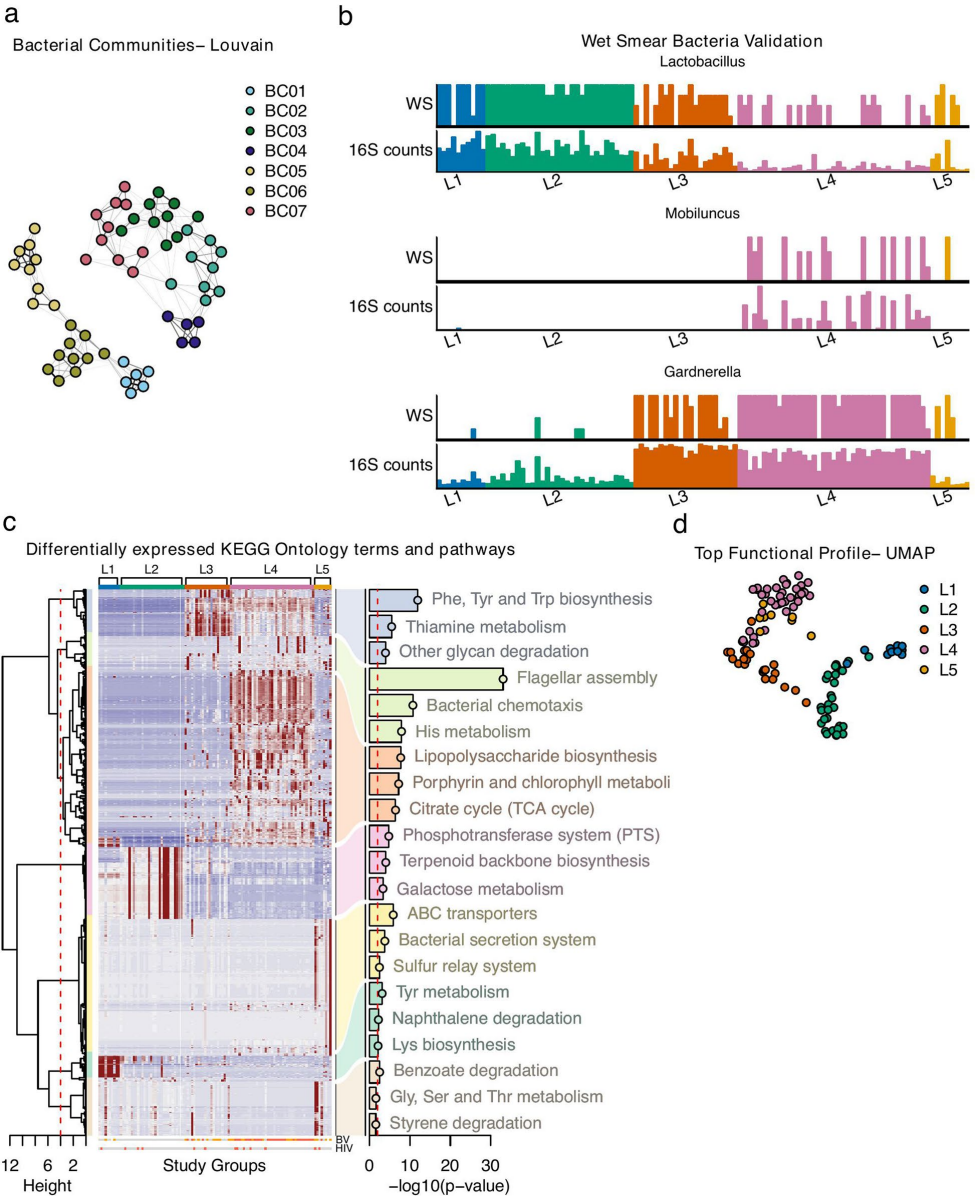
Identification of bacterial communities and functional profiles in the luminal samples. The luminal study samples were assessed for bacterial communities and functional profiles. **a** Bacterial community embedding of 5-SNN graph clustered using Louvain community detection algorithm based on luminal bacterial abundances. **b** Wet smear counts of the genera *Lactobacillus*, *Mobiluncus*, and *Gardnerella* with corresponding 16S read counts. **c** Differential expression analysis was applied to PICRUSt2 predicted KO terms across the five luminal study groups. Resulting significant (FDR < 1 × 10^−5^) KO terms were divided into seven modules by hierarchical agglomerative clustering using inverse Pearson’s correlation as distance measure and Ward’s method (“ward. D2”) for linkage. Enrichment analysis was performed on each module and the three most significant KEGG pathways were included in the heatmap. **d** Uniform Manifold Approximation (UMAP) of the predicted KO terms

Microbes at mucosal sites form sub-communities based on specific interactions between individual taxa. The inter-microbial relationships that define these communities can be inferred from the co-occurrence of taxa across multiple samples. We identified co-variant microbes across major taxa in the luminal samples that formed seven major bacterial communities (BCs) (Fig. 2a; Suppl. Table 1). Pathobionts such as *Pseudomonas* (BC05), *Ureaplasma*, and common gut bacteria, including *Streptococcus* and *Escherichia/Shigella* (BC06), showed a stronger co-occurrence with *Lactobacillus* (BC01) than with common BV-associated bacteria; *Atopobium* (BC02), *Gardnerella* (BC03), *Prevotella* (BC04), and *Porphyromonas* (BC07) (Fig. 2a). Quantification of the *Lactobacillus*, *Gardnerella*, and *Mobiluncus* morphotypes in the stained bacterial smears obtained at the time of sample collection showed a strong correlation with 16S rRNA V4 gene quantification (Fig. 2b). However, Gram-negative rods designated clinically as *Mobiluncus* morphotypes by Gram stain may partly represent BVAB1 [21]. *L. crispatus* and *L. iners* belonged to BC01, *Mobiluncus* to BC02, *Gardnerella* to BC03. A strong match is thus seen between the identified BCs and bacterial staining, suggesting a relatively simple clinical method for distinguishing the subgroups.

To determine whether the luminal microbiome impacts the metabolic profile of the cervix, we performed PICRUSt2 functional capacity estimates (Fig. 2c; Suppl. Table 3). These results align well with the luminal groups defined by bacterial taxonomic abundance as illustrated in a UMAP analysis (Fig. 2d). The *Lactobacillus* groups L1 and L2 showed the lowest enrichment for tryptophan biosynthesis, consistent with the ability of these bacteria to catabolize tryptophan but not produce it [22]. Also, these groups were enriched for galactose metabolism and terpenoid backbone biosynthesis genes, which are found predominantly in fermenting bacteria such as *Lactobacillus*. Functional analysis also distinguished L1 from L2 by lysine production genes, which are present in *L. crispatus* but nearly absent in *L. iners* [23]. Groups L3 and L4 were enriched for phenylalanine, tyrosine, and tryptophan biosynthesis, consistent with the ability of many *Gardnerella* strains to use these amino acids for biofilm formation. The L4 group also showed enrichment for lipopolysaccharide (LPS) biosynthesis, the citrate cycle, flagellar assembly, and bacterial chemotaxis. This is consistent with the abundance of LPS-producing, flagellated, Gram-negative *Prevotella* in L4. Thus, the functional capacity of bacteria in the cervicovaginal lumen environment differs drastically between groups and is closely linked to the microbiome composition.

### The ectocervical tissue-adherent microbiome is distinct from the luminal microbiome

**Fig. 3:**
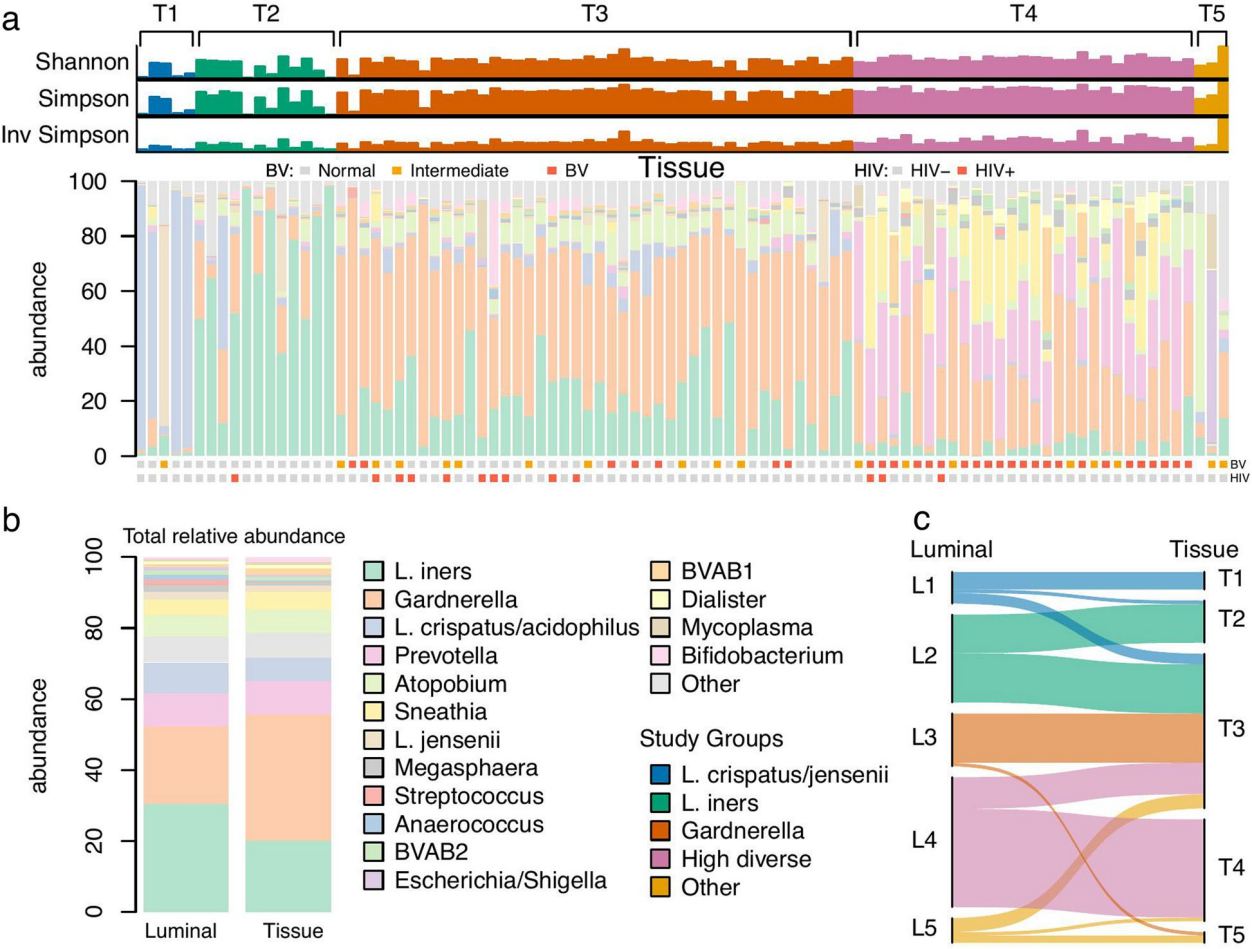
Identification of a distinct ectocervical tissue-adherent microbiome. Ectocervical tissue samples were assessed for presence of a tissue-adherent microbiome. **a** Bar plots of alpha diversity indices and taxonomy profiles for each individual tissue sample. Color-coded squares above the stacked bar plots show bacterial vaginosis (BV, binned Nugent’s scores) and HIV diagnosis, respectively. Gray: negative, orange: intermediate, red: positive BV; Gray: HIV seronegative, red: HIV seropositive. **b** Total relative abundance in the luminal and tissue microbiome datasets. All taxa with a total relative abundance < 0.55 are included in the “other” category. **c** Microbiome profile shift between luminal and tissue samples

Because tissue contact is important in host-microbe interactions, microbiomes may differ between the epithelial tissue surface and the corresponding lumen. To characterize the bacterial communities that adhere to the epithelium, we analyzed 93 ectocervical tissue samples from our study cohort (Fig. 3a; Suppl. Table 4). The most prevalent bacterial genera were *Gardnerella,* with a relative abundance of 36%, followed by *Lactobacillus* (29%, with *L. iners* representing 20% and *L. crispatus* 7%), *Prevotella* (10%), *Atopobium* (7%), and *Sneathia* (5%) (Fig. 3a,b; Suppl. Table 1). The tissue samples (named T for “tissue”) were divided into five predefined groups to correspond to the luminal counterparts; 5 samples with >50% abundance of *L. crispatus* and/or *L. jensenii* were assigned T1 (*L. crispatus*); 12 samples with >50% *Lactobacillus* (mainly *L. iners*) and <30% *Gardnerella* were assigned T2 (*L. iners*); 44 samples with >30% *Gardnerella* and <10% *Prevotella* were assigned T3 (*Gardnerella*); 29 samples with >10% *Prevotella* were assigned T4 (highly diverse); 3 samples with majority bacteria *Atopobium* (72%), *Gardnerella* (24%), and *Escherichia*/*Shigella* (63%) did not fit these thresholds and were assigned T5 (other) (Fig. 3).

All participants with tissue analysis also had characterization of luminal samples. In 60 of the 93 participants, the luminal and tissue microbiome groups corresponded well (5 women were categorized as *L.crispatus* in both L1 and T1, 11 women as *L. iners* in both L2 and T2, 14 women as *Gardnerella* in both L3 and T3, 28 women as highly diverse in both L4 and T4 and 2 women as other in both L5 and T5). A comparison between the paired luminal and tissue samples showed a higher alpha diversity in the tissue samples (Shannon and Simpson diversity *p*<0.05, inversed Simpson did not reach significance). Comparing the alpha diversity between each paired luminal and tissue group showed that all but the *L. crispatus* groups showed a significantly higher alpha diversity in the tissue group (Fig. 1a and Fig. 3a; Suppl. Table 1). *Gardnerella* and *Atopobium* were found in nearly all tissue samples, even in *Lactobacillus*-dominated samples (Suppl. Figure 1, Suppl. Figure 2). We next analyzed whether the luminal and tissue samples had a comparable microbiome composition, or a major shift as defined by belonging to another study group (Fig. 3c). Interestingly, almost half of the luminal samples that were categorized as L1 and L2 had a corresponding tissue sample categorized as T3. Women with a non-*Lactobacillus*-dominant microbiome were more likely to share a similar microbiome in their luminal and tissue samples; 93% of L3 were classified as T3 and 76% of L4 were classified as T4. A few genera (*Pelomonas*, *Bifidobacterium*, *Enterococcus*, *Janthinobacterium*, *Listeria*) were almost exclusively found in tissue-associated samples, while *Parvibacter* and *Rhodococcus* were almost exclusively found in luminal samples (Suppl. Figure 2). Furthermore, *Gardnerella* was present in almost all tissue samples.

### The luminal microbiome composition tightly associates with the host mucosal transcriptome

**Fig. 4:**
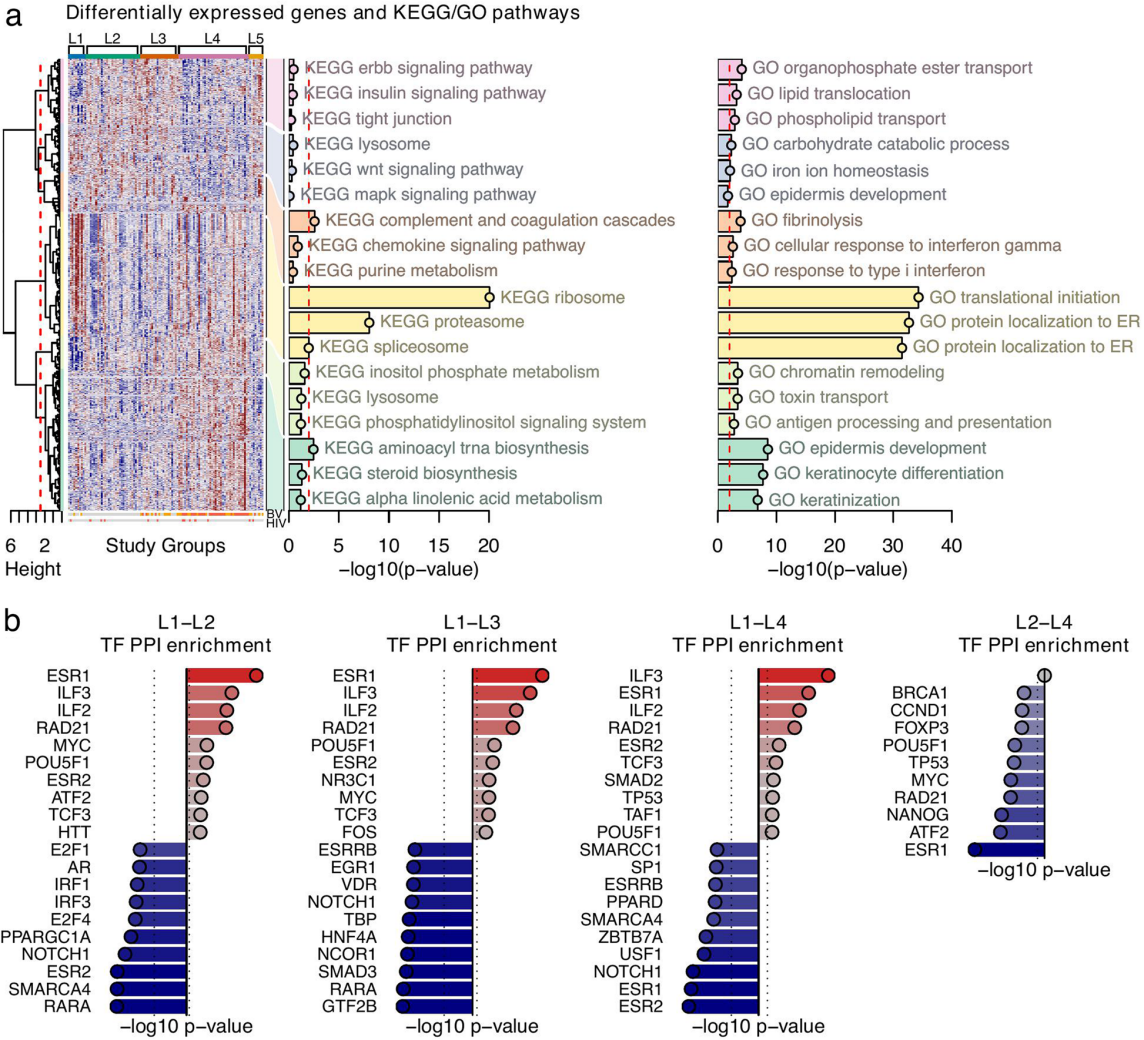
Characterization of the host transcriptome as stratified by the luminal microbiome study groups. The luminal samples were assessed for differential gene expression across the study groups. **a** Differential gene expression analysis was applied across the five luminal study groups. Significant DEGs (*p*-value < 0.01) were divided into six modules by hierarchical agglomerative clustering using inverse Pearson’s correlation as distance measure and Ward’s method (“ward. D2”) for linkage. Enrichment analysis was performed on each module using both the KEGG and GO databases. The three most significant terms were included in the heatmap. **b** Pairwise enrichment analysis of protein-protein interactions of transcription factors (TF-PPI). Top-10 up- and downregulated transcription factors with *p*-value < 0.01 were included in the bar plots

To determine the association of the composition of the luminal microbiome with the host transcriptional profile, we sequenced the mRNA of the ectocervical tissue samples. Among 15,435 protein-coding genes, a total of 868 were differentially expressed (*p* < 0.01) in a groupwise analysis across the L1–L5 groups after adjusting for possible confounding effects of DMPA use (*n* = 34) or HIV seropositivity (*n* = 13) (Suppl. Table 5); however, these factors did not affect the groups significantly (*p* > 0.05) (Table 1). Hierarchical clustering of the 868 differentially expressed genes (DEGs) identified six major gene modules with 127, 95, 75, 238, 64, and 269 genes. Modules 4 and 6 were associated with GO pathways that were significant after multiple comparison adjustments, while only module 4 associated significantly to KEGG pathways (false discovery rate, FDR < 0.05) (Fig. 4; Suppl. Table 6).

The significant DEGs, their associated GO and KEGG pathway, as well as transcription factor protein-protein interaction (TF-PPI) network analysis, are described in pairwise comparisons between the luminal groups as follows (Suppl. Table 5; Suppl. Table 7; Suppl. Table 8; summarized in Suppl. Figure 3A). Pairwise comparisons for L1 versus L2, L3, L4, and L5 showed 468, 413, 347, and 157 upregulated DEGs, respectively. These DEGs were associated with protein translation processes, which also came up significant in the groupwise analysis as shown in module 4 in the GO and KEGG pathway heatmaps (Fig. 4a). The high enrichment for the transcription factors ESR1, ILF3, and ILF2 in the L1 group suggested, among other effects, possible estrogen-associated gene regulation (Fig. 4b). Pairwise comparisons for L2 versus L1, L3, L4, and L5 showed 191, 51, 311, and 7 upregulated DEGs, respectively. None of these DEGs had any GO or KEGG associations, and TF-PPI analysis revealed enrichment only against L1 (RARA, SMARCA4, ESR2). Pairwise comparisons for L3 versus L1, L2, L4, and L5 showed 250 (GO pathway: “vacuolar transport”; TF-PPI: GTF2B, RARA, SMAD3), 49 (GO pathway: “cell division”), 221, and 26 upregulated DEGs, respectively. Pairwise comparisons focusing on L4 identified GO pathways associated with epithelial remodeling activity to be specifically enriched compared to L2 and L5. The L4 group also differed significantly from L1 (*n* = 349 DEGs) with GO associations to pathways involved in membrane budding and vesicle transport, which are required for intracellular trafficking. Comparisons between L4 and L2 (*n* = 577 DEGs) revealed GO associations to keratinization and cell division, immune mechanisms, toxin transport, and metabolic processes, as well as KEGG association to “aminoacyl tRNA biosynthesis” which is essential for protein synthesis. “Toxin transport” also defined L4 as compared to L3 (*n* = 189 DEGs) and L5 (*n* = 191 DEGs). For L4, TF-PPI analysis revealed the greatest enrichment for ESR2, ESR1, and NOTCH1 compared to L1, and ESR1 compared to L2. The average number of total DEGs (up- or downregulated) for each group across four pairwise comparisons was highest for L4 and L1 (577 and 557 DEGs) followed by L2 (415 DEGs), L3 (301 DEGs), and L5 (142 DEGs) (Suppl. Table 5).

In summary, L1 was mainly associated with genes upregulated for protein synthesis and transcription factors ESR1, ILF3, and ILF2. L4 was associated with genes upregulated for innate immunity, glucose catabolism, epithelial remodeling, and transcription factors ESR1, ESR2, and NOTCH1 compared to L2; membrane budding and ESR1, ATF2, and NANOG compared to L1; and toxin transport compared to L3. The transcriptional profiles of L2 and L3 were less distinguished with lower number of DEGs compared to L1 and L4. L2 showed less protein translation compared to L1 and less cell division compared to L3. L3 showed more vacuolar transport and GTF2B, RARA, and SMAD3 compared to L1 and more cell division compared to L2.

### The tissue-adherent microbiome is associated with fewer differentially expressed host genes compared with the luminal microbiome

**Fig. 5:**
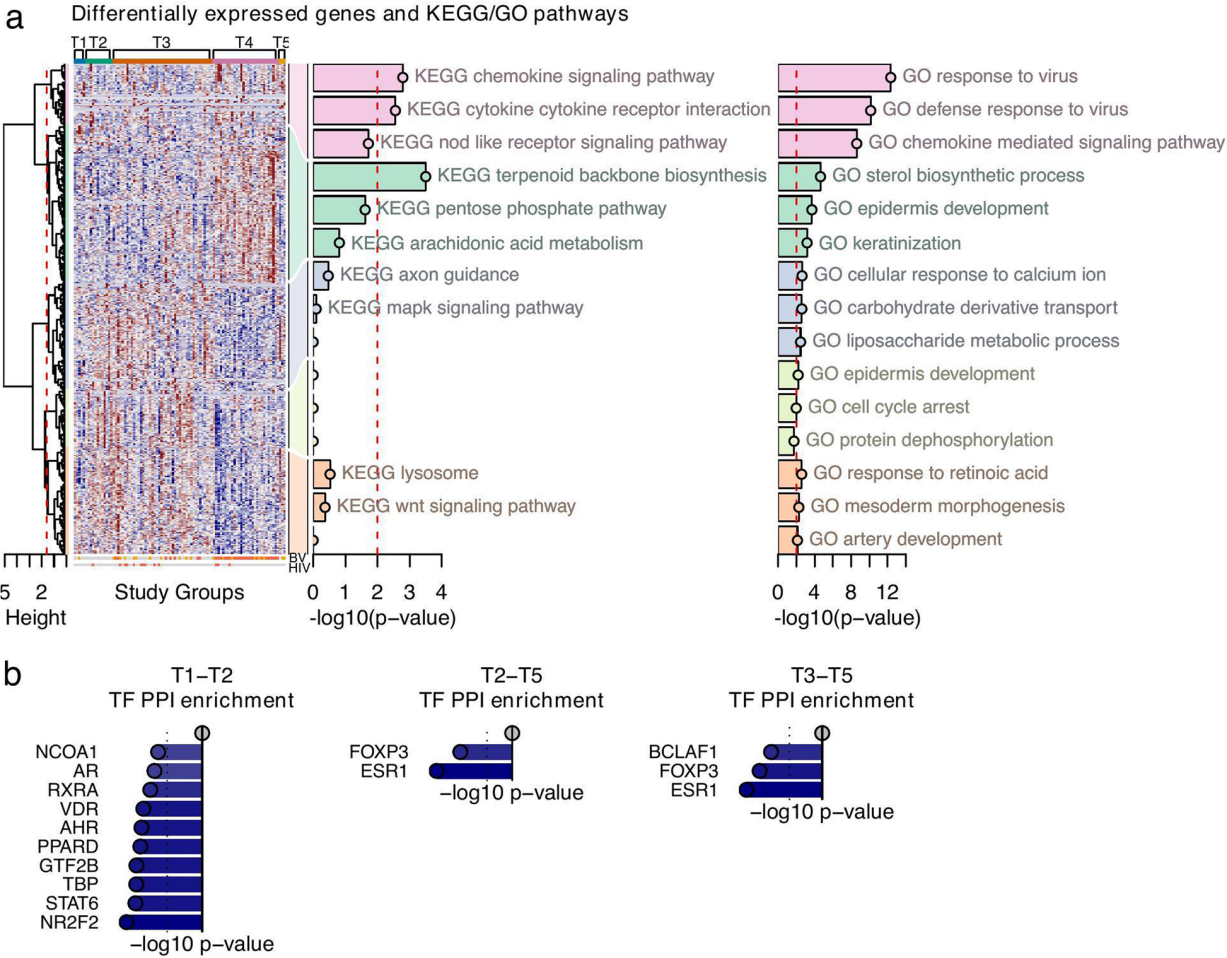
Characterization of the host transcriptome as stratified by the tissue-adherent microbiome study groups. The tissue samples were assessed for differential gene expression across the study groups. **a** Differential gene expression analysis was applied across the five tissue study groups. Significant DEGs (*p*-value < 0.01) were divided into five modules by hierarchical agglomerative clustering using inverse Pearson’s correlation as distance measure and Ward’s method (“ward. D2”) for linkage. Enrichment analysis was performed on each module using both the KEGG and GO databases. The three most significant terms were included in the heatmap. **d** Pairwise enrichment analysis of protein-protein interactions of transcription factors (TF-PPI). Top-10 up- and downregulated transcription factors with *p*-value < 0.01 were included in the bar plots

Analysis of the host transcriptomes for groups T1–T5 revealed fewer numbers of DEGs compared with groups L1–L5 (389 vs 868 DEGs; Suppl. Table 9, Suppl. Table 5). Hierarchical clustering of the 389 DEGs resulted in five gene modules with 49, 125, 85, 46, and 84 genes (Fig. 5; Suppl. Table 10). Module 1 in the heatmap was associated with GO and KEGG terms that included responses to viruses and a chemokine-mediated signaling pathway.

Pairwise comparisons of significant DEGs, associated GO and KEGG pathways, and TF-PPI between tissue groups are described as follows (Suppl. Table 9; Suppl. Table 11; Suppl. Table 12; summarized in Suppl. Figure 3B). In pairwise comparisons for T1, there were less than 100 DEGs per pair, and the upregulated DEGs were mainly involved in protein synthesis for T2, T3, and T5. The T1 group associated with the GO term “sterol biosynthesis” compared to group T3 and T5, and KEGG terms “ribosome” and “terpenoid backbone synthesis” compared to group T5. The 93 upregulated genes in T1 compared to T4 were not associated with any GO or KEGG pathways. The increase in genes associated with protein synthesis that was found for T1 was similar for L1. In pairwise comparisons for T2 and T3, there were less than 100 DEGs compared to the other tissue groups, except that T2 had 245 DEGs compared to T4, and T3 had 302 DEGs compared to T4. None of the upregulated DEGs in T2 and T3 had any significant GO or KEGG associations. In pairwise comparisons for T4, the number of upregulated DEGs was 57, 140, 226, and 37 for T1, T2, T3, and T5, respectively. The DEGs from the T4 vs T2 comparison were associated with the GO term “response to type I interferon,” and the T4 vs T3 comparison with immune responses, epithelial development, and response to toxic substances and to fatty acid derivative and arachidonic acid metabolism. For all pairwise comparisons across the T1–T5 groups, only the *L. iners* group (T2) had upregulated transcription factors compared to the *L. crispatus* group (T1) (including NR2F2, STAT6, TBP), and the T5 group showed upregulation of ESR1 and FOXP3 compared to the *L. iners* and *Gardnerella* groups (Fig. 5b, Suppl. Table 12).

*L. iners* has either protective or pathogenic effects on the cervicovaginal epithelium [24]. Since women in the L2 group (*L. iners*) grouped either to T2 (“L2T2,” *n* = 12) or T3 (“L2T3,” *n* = 17), we examined their transcriptome profiles for possible pathogenic effects associated with either group. A total of 34 DEGs were upregulated and 17 DEGs were downregulated in L2T2 vs L2T3, but none were significantly associated with GO and KEGG terms or pathways (FDR > 0.05) (Suppl. Table 13). However, the upregulated DEGs in L2T2 with the greatest fold change were genes for keratinization proteins KRT2 and KRT3, for cell adhesion protein LGALS4, and for the inflammatory protein IL17A, all proteins that strengthen the epithelial barrier.

In summary, based on the number of DEGs and associated pathways, the tissue-adherent microbiome had an impact on host transcriptome profiles, although less than the luminal microbiome. These responses were overall comparable in function between the sample types.

### Microbial drivers of the host mucosal transcriptome

We next examined the role of specific bacterial genera from the luminal microbiome dataset on host ectocervical gene expression to identify possible microbial drivers of host transcriptional patterns (Suppl. Figure 4). Host arachidonic and linoleic acid metabolism was significantly associated with a group of BV-associated taxa, including *Atopobium*, *Gardnerella*, BVAB2, *Megasphaera*, *Prevotella*, *Sneathia*, BVAB3, and *Mobiluncus* (highlighted in yellow in Suppl. Figure 4). “Antigen processing and presentation” and several immune signaling pathways correlated with pathogenic bacteria including *Mycoplasma*, *Streptococcus, Escherichia*/*Shigella*, and *Pseudomonas* (highlighted in purple in Suppl. Figure 4). A heatmap for the tissue microbiome dataset (Suppl. Figure 5) revealed arachidonic and linoleic acid metabolism as significantly associated with BV pathogens, including *Prevotella*, BVAB2, BVAB3, *Megasphaera*, *Sneathia*, and others (highlighted in yellow). This is similar to the upregulation of genes for arachidonic acid metabolism in the T4 group in the KEGG gene set enrichment (Fig. 5). Immune signaling pathways (including TCR, TLR, JAK-STAT, and chemokine signaling pathways) correlated with *Escherichia/Shigella*, *Enterobacter*, *Pseudomonas*, C*ampylobacter*, and other gastrointestinal microbiota (highlighted in orange in Suppl. Figure 5). “Antigen processing and presentation” and other immune activation pathways were also associated with the prevalent, tissue-adherent pathogenic bacteria *Mycoplasma*, *Prevotella*, and *Dialister* (highlighted in green in Suppl. Figure 5). In summary, our multimodal integrative analysis of gene expression associated certain microbes with changes in the host tissue and with specific host functions. *L. crispatus* and *L. iners* were not associated with a distinct pattern of gene expression in either the luminal or the tissue-adherent microbiome data sets. However, BV-associated bacteria associated with arachidonic and linoleic acid metabolism as well as immune signaling pathways in both data sets.

### The luminal and tissue-adherent microbiome communities correlate with cervicovaginal protein levels

**Fig. 6:**
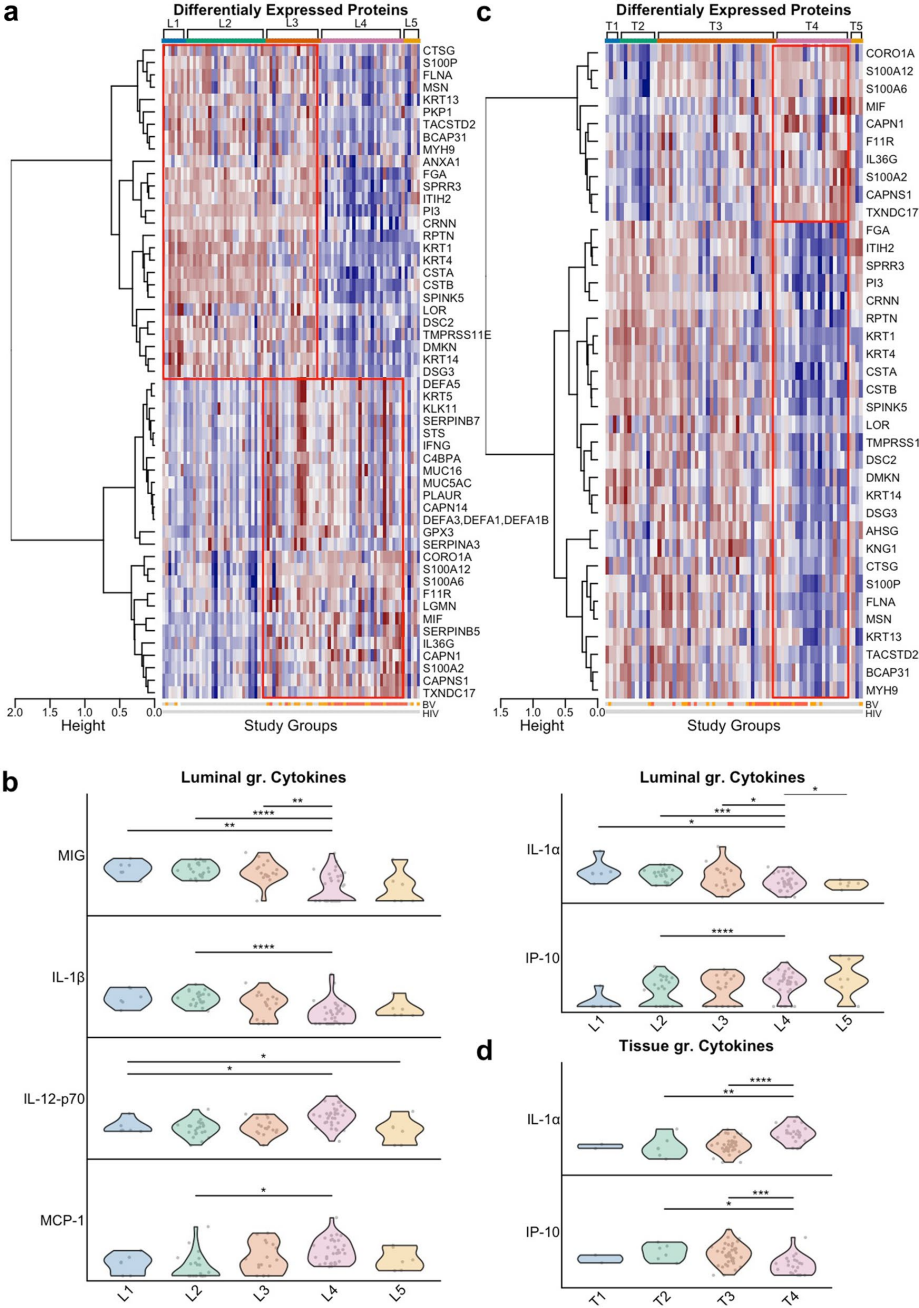
Characterization of the host protein profile as stratified by the luminal and tissue-adherent microbiome study groups. All study samples were assessed for significant differences in protein levels across the study groups. **a,b** The five luminal study groups and **c,d** the five corresponding tissue groups. **a, c** Proteins with a *p*-value > 0.05 were omitted from the heatmap. Proteins with significantly different levels across the groups were clustered by hierarchical agglomerative clustering using inverse Pearson’s correlation as distance measure and Ward’s method (“ward. D2”) for linkage. Color-coded rows below the heatmaps show clinical diagnosis of bacterial vaginosis (BV, binned Nugent’s scores) and HIV diagnosis, respectively. Color coding for BV: Gray: negative, orange: intermediate, red: positive; and for HIV: Gray: HIV seronegative, red: HIV seropositive. **b, d** Violin plots of log_2_ transformed cytokine levels across the luminal and tissue study groups (as indicated on the *x*-axis), respectively. The asterisk and lines indicate statistically significant results of Dunn’s test with Benjamini Hochberg’s correction analysis. Adjusted *p*-values: * < 0.05, ** < 0.01, *** < 0.001, **** < 0.0001

After analyzing the correlations between the luminal and tissue-adherent microbiota and the host tissue transcriptome, we examined the correlation with specific protein levels in corresponding cervicovaginal lavage samples. We analyzed 74 proteins, chosen for their relevance to the genital epithelial barrier and immune regulatory activities (Suppl. Table 14). Based on availability and HIV seronegative status, 84 luminal samples were analyzed from the total collection (L1, *n* = 8; L2, *n* = 26; L3, *n* = 18; L4, *n* = 27; L5, *n* = 5) (Suppl. Table 15). Among the 74 proteins, 53 had significantly different levels when comparing all luminal study groups (FDR adjusted *p*-value < 0.05) (Fig. 6a; Suppl. Table 16). Hierarchical clustering of these proteins identified (tree cut at height=1) two clusters, one cluster of 27 proteins with higher levels in L1–L3 compared to L4, and one cluster of 26 proteins with lower levels in L1-L2 compared to L3–L4. While the same protein may be assigned multiple functions, upregulated activities for the L1–L3 groups included cytoskeleton modification (FLNA, MSN, MYH9), wound repair (FGA), and protease inhibition (ITIH2, CSTA, CSTB, PI3, SPINK5). A more detailed pairwise comparison (FDR < 0.05) confirmed that the levels of most of these proteins were higher in L1–L3 than in L4. The pairwise comparisons also revealed that some of these proteins were more upregulated in L1 than in L3 (KRT1, KRT4, KRT14), and in L2 than in L3 (KRT1, KRT4, KRT13, CSTA, CSTB, SPINK5). The second block of proteins in the heatmap included proteins involved in cell proliferation (SERPINB5, CAPN1) and tissue regeneration (S100A2), as well as pro-inflammatory proteins (IL36G, MIF, S100A12). Pairwise comparisons revealed that many of the proteins in the second block showed higher levels in L3–L4 compared to L1–L2. In addition, the two *Lactobacillus-*dominated groups L1 and L2 showed no significant difference in protein levels. Among 16 selected cytokines measured in the luminal samples (total = 85; L1, *n* = 7; L2, *n* = 23; L3, *n* = 16; L4, *n* = 33; L5, *n* = 6), six cytokines had significantly different protein levels between the groups (adjusted FDR < 0.05) (Fig. 6b; Suppl. Table 14). While L1 and L2 had comparable cytokine levels, L4 had a significantly higher pro-inflammatory cytokine response (high IL-1α, IL-1β, IL-12-p70, and low IP-10, MIG, and MCP-1).

In comparisons between the levels of secreted protein and the tissue-adherent microbiome groups (Fig. 6c,d; Suppl. Table 17), 37 proteins were significantly different in the groupwise analysis and clustered in two blocks of 10 and 27 proteins respectively. We found that the two *Lactobacillus*-dominated groups, T1 and T2, were similarly associated with increased levels of protease inhibitors and epithelial barrier stability proteins (second cluster of 27 proteins including SPRR3, desmosomes, and keratin family members) and distinct from T4. The large majority of these 27 proteins were also increased in the *Gardnerella*-dominated T3 group compared to the highly diverse T4 group. The smaller cluster of 10 proteins were increased in the T4 group, which was defined by the upregulation of proteins from the S100 family, which has antibacterial and tissue regeneration roles, the protease CAPN1, and the inflammatory cytokine MIF. The T4 group also had higher IL-1α activity and lower IP-10 activity than T2 and T3. Overall, pairwise comparisons between the luminal microbiome groups correlated with more changes in protein levels than between the tissue-adherent microbiome groups (total number of significantly different proteins in all ten pairwise comparisons were 174 versus 85, respectively) (Suppl. Table 16 and Suppl. Table 17). Similarly, for the luminal and tissue groups, we observed 6 vs. 2 significantly different cytokine levels, respectively (Fig. 6, Suppl. Table 14).

Analysis of different protein levels from groups with *L. iners*-dominated luminal and tissue-adherent microbiomes (L2 and T2) versus those with heterogenous microbiomes (L2 and T3) showed no significant differences between the L2T2 and L2T3 groups (Suppl. Table 13).

## Discussion

We report characterization of a tissue-adherent microbiome in human ectocervical biopsies that had a distinct composition compared with its cervicovaginal luminal counterpart. The most prevalent bacterial genus in the tissue-adherent samples was *Gardnerella* with a total relative abundance of 36%, followed by *Lactobacillus* (*L. iners*, 20%; *L. crispatus*, 7%), *Prevotella*, *Atopobium*, and *Sneathia*. In the cervicovaginal luminal samples, *Lactobacillus* was more abundant than *Gardnerella*. While the tissue-adherent microbiome in the ectocervix has not been well-characterized previously, the overall relative abundance of luminal bacteria resembled other adult sub-Saharan African cohorts [12, 25–28]. In contrast, in Caucasian populations, *L. crispatus* is the dominant cervicovaginal strain [10]. Bacterial alpha diversity was higher in the tissue-adherent samples compared to the luminal samples, similar to the results for tissue and luminal samples from the human endometrium [29]. *L. crispatus*, *L. iners*, *Gardnerella*, and *Atopobium* were present in nearly all tissue samples. As the 16S rRNA gene sequencing was done on intact cervical tissue biopsies collected after cervicovaginal lavage treatment, these bacteria are likely to represent the tissue-adherent population. Loosely attached bacteria would likely have appeared in the lavage (here named “luminal”) samples. The segregation of microbiomes was further supported by the differences in the dominance of *Gardnerella* and *L. iners* between tissue and luminal samples.

*Gardnerella* and *Atopobium* form vaginal biofilms that remain as a bacterial reservoir despite antibiotic treatment for BV [30]. The ectocervical tissue-adherent microbiome can possibly form a biofilm that seeds the luminal microbiome with BV-associated bacteria upon changes in the local environment that disturbs the *Lactobacillus* abundance (i.e., after antibiotic treatment or during menses, sex, or douching) [31]. We showed recently that *L. iners*-dominated, but not *L. crispatus*-dominated, communities can transition to highly diverse bacterial communities with a high abundance of *Gardnerella* and *Atopobium* [32]. Here, we found that 56% of the samples with an *L. iners*-dominated luminal microbiome had a *Gardnerella*-dominated tissue-adherent microbiome. The latter community could provide the source for these microbiome transitions. Biofilms in human cervical tissue may also impact other cervical infections such as HPV entry and replication, but they are poorly characterized [33]. For gonorrhea, infection of the endocervix involves biofilms [34, 35], and the ectocervix contains tissue-adherent anaerobic *Lactobacilli* [18]. *Gardnerella* species adhere strongly to vaginal cells, colonize the vaginal epithelium, and form a biofilm which serves as a scaffold where other species can attach and even resist antibiotic treatment for BV [36–41]. While some BV-associated species lack key virulence traits [42], we found that samples with higher abundances of *Prevotella*, *Sneathia,* BVAB1, and BVAB2 coexisted with *Gardnerella* but were not present in *Lactobacillus*-dominated tissue samples. Little is known about the endogenous bacterial microbiome that may extend deeper into the cervicovaginal epithelium and submucosal tissue in healthy women. In our study population, the tissue-adherent microbiome includes some gut-associated bacteria and pathobionts capable of causing severe genital epithelial damage and inflammation despite their low abundance [43, 44]. It is likely that pathogenic bacteria can invade more deeply into the cervicovaginal epithelium under unfavorable host conditions. For example, histopathological vaginal epithelial lesions are found when *L. iners*, *Gardnerella*, and *Atopobium* accompany vaginal yeast infections [45]. Despite its association with protection from vaginal infections, *L. crispatus* was also shown to invade the vaginal epithelium during a yeast infection. It seems unlikely that the intact multilayered cervicovaginal squamous epithelium that we studied here allows bacteria to enter the submucosa below the basal cell membrane. It would be interesting to see if the composition of the tissue-adherent microbiome in these Kenyan female sex workers are comparable to that of other non-sex-working cohorts with a higher prevalence of luminal *L. crispatus*, such as Kenyan school girls [46], and women of some other ethnicities [10].

Although the microbiome in cervicovaginal fluids is known to affect host genital mucosal immune responses [11, 12, 43], we provide new insights on the associations between the tissue-adherent microbiome and the cervical tissue function by integrating microbiome sequencing data with host transcriptional profiling. The clinical samples were divided into groups based on the dominant bacteria: *L. crispatus*, *L. iners*, *Gardnerella*, “highly diverse,” and “others.” Of the 15,435 human genes that were identified, approximately 5% of the genes differed between the luminal groups and about 2.5% between the tissue-adherent groups. This difference may reflect a higher bacterial load in the lumen, or the luminal bacteria may have better receptor-signaling activity. The tissue-adherent bacteria may be less metabolically and immunologically active, or partly represent bacterial DNA reminiscence, if capsulated into biofilm formations. The host genes associated with the luminal *L. crispatus* group were found to be involved with processes for protein translation and innate immune responses. Unlike many pathogenic anaerobes, *Lactobacilli* and the lactic acid they produce promote antimicrobial defenses without inducing immune-mediated inflammation. Lactic acid also promotes tight junction protein expression and thereby contributes to epithelial barrier integrity [47]. Enrichment analysis for transcription factors in the *Lactobacillus* groups revealed an increase in estrogen-associated regulation (ESR1). High levels of estrogen promote a *Lactobacillus*-dominated microbiota and *Lactobacillus* spp. increase vaginal glycogen deposition and stimulate lysis of infected epithelial cells. Increases in the transcription factors ILF2 and ILF3 correlated strongly with the *L. crispatus* group and they may account for changes in the innate immune response. The *L. iners-*dominated and *Gardnerella-*dominated groups showed fewer differences in their transcriptional profiles than the other groups. This may be associated to a different transcriptional activity in these bacteria when co-habiting with BV-associated bacteria [19, 20]. The samples from the highly diverse group were, however, associated with the upregulation of genes involved in epithelial remodeling activities, such as epithelial development and keratinocyte differentiation. This is consistent with a previous report that women with a BV-associated microbiome have increased vaginal epithelial shedding compared to women with a *Lactobacillus*-dominated microbiome [48]. Samples from the highly diverse group were associated with increased transcription of ESR1 and ESR2 for estrogen regulation. In comparison with the *L. iners* group, the highly diverse group showed increased gene expression that translated to innate immune response pathways, possibly by the upregulated transcription factor NOTCH1. Vaginal communities dominated by anaerobes are associated with increased pro-inflammatory responses compared to those dominated by *L. crispatus* [11, 43]. The anaerobes in the highly diverse group may account for the gene enrichment pathway analysis results showing pathogen-stimulated inflammatory response and increased activity for toxin transport.

A cervicovaginal microbiota dominated by *L. iners* has been associated with both vaginal health and vaginal dysbiosis, and the latter is associated with an increased vaginal pH and the production of species-specific virulence factors [24, 49–51]. We hypothesized that these disparate effects could be intrinsic to various subtypes of *L. iners* and/or influenced by differential gene expression by its association with *Gardnerella*-containing biofilms. However, the transcriptional and protein profiles of the samples from the luminal *L. iners* group were strikingly similar whether they paired with the tissue-adherent microbiome dominated by *L. iners* or dominated by *Gardnerella*. We also compared transcriptional profiles based on bacterial genera (or species level for *Lactobacillus* and BVAB) in addition to our analysis of transcriptional profiles for the subject-based microbiome groups. For the luminal microbiome data set, a group of bacteria including *Atopobium*, *Gardnerella*, BVAB2, *Megasphaera*, *Prevotella*, and *Sneathia* were significantly associated with arachidonic and linoleic acid metabolism. These fatty acids respond to irritation by stimulating epithelial growth and serve as mediators of inflammation. Higher levels of arachidonic acid catabolite 12-hydroxyeicosatetraenoic acid have previously been shown in women with BV [52]. Another group of bacteria that can be pathogenic, including *Fusobacterium*, *Paraprevotella*, *Streptococcus*, and *Escherichia/Shigella* [44], correlated with immune activation pathways. A similar pattern was seen for the tissue-associated microbiome.

In addition to the cervical tissue transcriptional profiles, we measured protein levels in the corresponding cervicovaginal lavages and compared them to the luminal and tissue-adherent microbiomes. In general, the protein profiles aligned with the results from the transcriptional profiles, including the greater impact of the luminal versus the tissue-adherent microbiome on host responses. Protein levels for the two *Lactobacillus*-dominated luminal and tissue-adherent study groups were indistinguishable and included proteins with anti-inflammatory and epithelial stabilizing properties. The *Gardnerella*-dominated luminal group included more proteins with pro-inflammatory activity than the *Lactobacillus* groups. The protein profiles for the highly diverse groups included protease-rich, pro-inflammatory, and cell proliferative proteins. Proteome changes related to inflammation and loss of epithelial integrity have also been seen in other studies on non-*Lactobacillus*-dominated genital secretions [11, 43, 53].

Our study has limitations including the lack of clinical data on current HPV and HSV-2 infections that may influence the cervical mucosa. Gene sequencing provided information at only the genus level for most bacteria, limiting our ability to identify, in detail, the bacterial drivers of host responses. Identification of the host transcriptome was performed on bulk tissue samples that precluded evaluation at the single-cell level. Whole-genome DNA and RNA sequencing of the microbiomes could help to determine whether different bacterial genes are expressed, leading to tissue attachment or biofilm formation for only the tissue-associated bacteria. Identification of bacterial proteins in the tissue samples or imaging analysis of tissue sections could confirm biofilm formation. Nevertheless, the unique samples representing a highly relevant clinical female sex-working cohort and the experimental multi-omics approach we used revealed a distinct tissue-adherent microbiome. This community, along with the luminal microbiome, correlated with host gene expression and secreted proteins levels. Understanding the molecular pathways associated with mucosal host-microbial interactions in the lower female genital tract can help in the prevention and treatment of adverse reproductive events and sexually transmitted infections.

## Conclusions

While the microbiome composition in the human cervicovaginal tract has been defined, the presence and impact of a tissue-adherent ectocervical microbiota remain incompletely understood. Here, we characterized paired luminal and ectocervical tissue samples collected from a clinically well-characterized cohort of Kenyan sex-working women. Tissue-adherent bacterial communities were identified in all individuals. These communities were partly distinct from the luminal microbiota with regard to composition and correlation with host gene expression and cervicovaginal protein levels. The observed high abundance of *Gardnerella* in the tissue-adherent communities could possibly explain previous observations that *L. iners* dominant luminal communities have a high probability of transitioning to highly diverse bacterial communities, including *Gardnerella*. The ectocervical tissue-adherent microbiota may even seed the lumen with less optimal, non-*Lactobacillus*, bacteria. This could contribute to the high recurrency rate of BV following antimicrobial treatment. The present characterization of the female genital tract microbiome from different cervicovaginal compartments, together with detailed analyses of host-related molecular pathways, contributes to the understanding of women’s reproductive and sexual health.

## Methods

### Study subjects

This cross-sectional study included paired cervicovaginal lavage (luminal) and ectocervical tissue samples from women included in the Pumwani Sex Worker Cohort in Nairobi, Kenya [54, 55]. The samples were collected from 2013 to 2016. All subjects answered a demographic and behavioral questionnaire at the time of inclusion in the study and the study visit. To roughly standardize for sex hormone status, samples from women using DMPA were taken during a 4–8 week’s period following injection. For women who were not using hormonal contraceptives, samples were taken during the estimated follicular phase of the menstrual cycle, based on the reported number of days since their last menstrual period. In addition, plasma estradiol (E2) and progesterone (P4) levels were measured using electrochemiluminescence immunoassays (Roche Diagnostics) at the accredited Karolinska University Laboratory, Stockholm, Sweden. The limits of detection for E2 and P4 were 22 pg/mL and 0.05 ng/mL, respectively. Levels below the detection limit were assigned these values for statistical purposes. HIV serology was assessed using a rapid test (Determine, Inverness Medical, Japan). Bacterial vaginosis was defined by the Nugent score based on Gram-stained smears [54]. *Treponema pallidum* was detected with a serological test (Macro-Vue Rapid Plasma Reagin test, Becton Dickinson, Franklin Lakes, NJ, USA) and *Trichomonas vaginalis* was diagnosed by wet smear microscopy. *Neisseria gonorrhoeae* and *Chlamydia trachomatis* were detected in urine samples by PCR (Roche Amplicor, Pleasanton, New Jersey, USA).

### Sample collection

Cervicovaginal lavage samples were collected as previously described [54]. Briefly, 2 mL of sterile phosphate-buffered saline (PBS) was flushed into the vaginal cavity and collected from the posterior fornix region. Samples on ice were transported to the laboratory, centrifuged to separate mucus and cell debris (the “pellet”) from the lavage supernatant, and RNAlater was added to the pellets (QIAGEN, Valencia, CA). The supernatants and pellets were stored at −80°C. Plasma was separated from blood samples and both were stored at −80°C. A trained gynecologist collected two 3-mm^3^ biopsies from the superior portion of the ectocervix using Schubert biopsy forceps (model ER058R, Aseculap, Germany). One biopsy was immediately frozen in liquid nitrogen for immunofluorescence staining, one was placed in RNAlater, and both were stored at −80°C. The women agreed not to have unprotected sex for a 4-week study period, including 2 week’s post-biopsy and were compensated for the loss of income during this period. Participants were scheduled to return to the clinic 3 day’s post-biopsy for examination by the gynecologist to ensure ectocervical healing. The planned procedures for the sample collection and clinical follow-up have been published [54].

### Microbial 16S rRNA gene sequencing

DNA and RNA were extracted from the ectocervical tissue biopsies in RLT Plus Lysis Buffer (QIAGEN) by homogenization using a TissueLyser II machine (QIAGEN) and purified using the AllPrep DNA/RNA Mini Kit (QIAGEN) and QIAcube Connect (QIAGEN). DNA was further purified with the DNeasy PowerClean Pro Cleanup Kit (QIAGEN) for 16S rRNA gene sequencing. Nucleic acids from the lavage pellets were extracted using a phenol-chloroform protocol [56], with RNAlater storage solution being removed by spinning down and washing with 1000 μL of PBS prior to bead beating. The 16S rRNA V4 gene region was amplified using the 515F/806R primer set and sequenced using forward reads on Illumina MiSeq as previously described [56]. The 515F/806R primer set was used to ensure efficient amplification of *Bifidobacteriaceae* (including *Gardnerella*). ImageJ (v2.0.0) was used to measure the intensity of amplicons on the gel electrophoresis image. Sample band intensities were normalized against the DNA ladder (NE Biolabs, USA), and volume corresponding to 20 ng of each sample was added to the library pool. Besides luminal samples, two extraction controls and two amplification controls were included (purified water) with a total of 180, 260, 612, and 1392 reads respectively. The gene amplification aimed for a sequencing depth of 40,000 reads per luminal sample. Rarefaction curves showed by a flattening of the curve (i.e., few additional operational taxonomic units [OTUs]) that we had achieved sufficient read depth for all luminal samples (Suppl. Figure 6a). As the tissue samples contained large amounts of human DNA, the bacterial sequencing counts were relatively low as noted in a previous study [57], with a sequencing depth of >2500 reads in all but 16 samples. However, rarefaction curves indicated that all samples were suitable for taxonomic assignment (Suppl. Figure 6b). Besides tissue samples, three extraction controls and one amplification control (purified water) were included with a total of 10, 26, 101, and 49 reads, respectively. In addition, two positive control samples were included (ZymoBIOMICS Microbial Community Standard II (Log Distribution), Zymo Research, USA) (Suppl Table 1).

Sequences were demultiplexed using QIIME, followed by quality control and taxonomy assignment using Dada2 package (R Studio v.3.6.3) and the RDP 16S rRNA gene operational taxonomic unit (OTU) reference database. The taxonomic categories were refined by BLAST [58] searches of any unassigned sequences in the vaginal-specific database OptiVag v.1.0 [59]. This sequencing method has limited species resolution and thus search criteria were set to 99.5% identity and 95% coverage [60]. BVBA1–BVBA3 sequences were verified using a local database based on published sequences [61–63]. Sequencing depth was evaluated with rarefaction plots made with the rarecurve function from the Vegan package v. 2.5.6 [64]. Taxa with >3 counts in at least one sample were included. The microbiome data were normalized by total-sum scaling (TSS) for alpha diversity analysis as well as log_2_[CP1K+1] transformation before experimental analysis. The microbiome was characterized at the genus level, except for the *Lactobacilli* and BVAB1– BVAB13, which were characterized at the species level.

### Microbiome data

Alpha diversity was calculated with the vegan package v. 2.5.6 [64]. The Nearest Neighbor Search function (nn2) from the RANN package v. 2.6.1 [65] was used to construct a tree graph of study participants (Fig. 1c) and bacterial communities (Fig. 2a), using *k* nearest neighbor’s value of 5 and 12, respectively. The trees were pruned based on the Jaccard index before applying the Louvain clustering function from the igraph package v. 1.2.6 [66].. Heatmaps showing the functional association of microbiome datasets with the expression profiles from the RNA-seq dataset were constructed from bacterial abundance (of 146 taxa present in at least two samples), correlated with the gene expression of the top 5000 highly variable genes from the RNA-seq dataset, generating a correlation matrix between bacteria and genes. Then genes were ranked according to the correlation score for each bacterium. Gene set enrichment analysis (GSEA) was performed on each list of genes using the KEGG gene annotation database [67]. This, in turn, resulted in a matrix associating every bacterium with every KEGG process in the tissue. The heatmap shows the normalized enrichment score (NES) with a *p*-value < 0.05. Only bacterium and pathways with at least 10 significant NES scores were included in the heatmap. Differential bacterial abundance across luminal and tissue datasets was identified by the Mann-Whitney *U* test. Bacteria with log2FC above 0.25 and *p*-value < 0.01 were considered significant and were sorted by the highest expression values.

### Definition of study groups based on bacterial composition

The luminal samples were divided into five study groups based on their bacterial composition. The supervised categorization was based on our previous South African study cohort [9], with minor modifications as follows. Samples with a *Lactobacillus*-dominated microbiota were divided into two groups, categorized as L1 (> 80% *L. crispatus* and/or *L. jensenii*) and L2 (> 80% *Lactobacillus* spp.). Samples with *Gardnerella* (>10%) and low *Prevotella* (<5%) were assigned to L3, and samples with *Prevotella* > 5% were assigned to group L4. Samples that did not fit any of these categories were assigned to group L5. The tissue-associated microbiome showed a slightly different composition than the luminal samples with higher levels of *Gardnerella* and *Atopobium* across all samples. The abundance threshold for different genera was thus adjusted moderately when dividing the tissue samples into five study groups. Samples dominated by *Lactobacillus* were either categorized as T1 (> 50% *L. crispatus* and/or *L. jensenii*) or T2 (> 50% *Lactobacillus* spp*.* and *< 30% Gardnerella*). Samples with a high *Gardnerella* abundance (> 30% *Gardnerella*) and low *Prevotella* (< 10%) were categorized as T3. Samples with *Prevotella* (> 10%) were categorized as T4. Samples that did not fit any of these categories were assigned to group T5.

### Preparation of tissues, RNA sequencing (RNA-seq) analysis, dimensionality reduction analysis, and functional annotations

RNA from the RNAlater preserved fresh frozen ectocervical biopsies was isolated and purified with AllPrep DNA/RNA Mini Kit (QIAGEN, Hilden, Germany) and by QIAcube Connect (QIAGEN). Thawed biopsies were placed in RLT Plus Lysis Buffer (QIAGEN) and homogenized using a TissueLyser II machine (QIAGEN). RNA integrity number (RIN) was assessed by the Agilent 2200 TapeStation System (Agilent Technologies, Santa Clara, CA, USA). The TruSeq mRNA-Seq Library Prep Kit (Illumina, San Diego, CA, USA) protocol was used for poly-A enrichment, fragmentation, PCR amplification, barcoding, and sequencing with NextSeq 550 (Illumina, San Diego, CA, USA). Base-calling and de-multiplexing were performed with the bcl2fastq program (Illumina) resulting in single-end 75-bp reads. The STAR (Spliced Transcripts Alignment to a Reference) alignment program was used to map reads to annotated exons using data from UCSC (University of California Santa Cruz, Santa Cruz, CA, USA) genome browser (http://genome.ucsc.edu/ (accessed on February 15, 2021)). The program was run in R v. 3.6.0 [68] /Bioconductor v. 3.9 (BiocManager 1.30.4) [69]. Further data analysis was also done in the R. Read counts were TMM normalized with the edgeR package v. 3.28.0 [70], and genes with an absolute read count < 5 in at least three samples were removed. DEGs were calculated using a negative binomial generalized linear model (glm) from the EdgeR package [70]. EdgeR is specialized for complex experiments involving multiple treatment conditions and can block variables while still accounting for biological variation. HIV status and use of the DMPA contraception were included as blocking variables in the model. DEGs and enrichment analysis were performed (1) across all study groups and (2) as pairwise comparisons. Genes were considered differentially expressed for *p*-values < 0.01 across all groups, with a false discovery rate (FDR) < 0.05 in the pairwise comparisons. Downstream enrichment analysis included genes with *p*-values < 0.01 in both cases (i.e., across groups and pairwise, respectively). DEGs across all groups were split into gene modules identified by inverse Pearson correlation as the distance for hierarchical agglomerative clustering with Ward’s method (“ward. D2”). Functional gene annotation was performed on each gene module individually using the Gene Ontology (GO_Biological_Process_2017) [71, 72] and the Kyoto Encyclopedia of Genes and Genomes (KEGG_2016) [73–75] libraries with a self-written enrichment function. The analysis of protein-protein interactions of transcription factors (TF-PPI) [76, 77] used the enrichR package [76].

### Prediction of functional pathways by PICRUSt2

We used PICRUSt2 (Phylogenetic Investigation of Communities of Unobserved States) [78] to predict functional KEGG pathways [73–75]. The full pipeline was run using default settings on the luminal sample data sets. Amplicon sequence variants (ASVs) with five or fewer reads in three or fewer samples were removed before running PICRUSt2 since rare ASVs can add noise to the prediction result. All samples had sufficient sequencing depth (>10,000 reads) for inclusion in the analysis. The resulting file (pred_metagenome_unstrat.tsv), containing the predicted KEGG orthology (KO) terms normalized to the predicted 16S rRNA gene abundances, was used for group comparisons. The 5530 KO terms were assigned to 306 KEGG pathways using the picrust KEGG_pathways_to_KO.tsv file. The edgeR [70] package in R was used to perform a Genewise Negative Binomial Generalized Linear Models on TMM normalized data. *P*-values were calculated with the topTags function and adjusted with Benjamin Hochberg correction. Top KO terms were used for UMAP unsupervised dimensionality reduction analysis (uwot package v. 0.1.10) [79] which was performed using the normalized count per million. Only KO terms with an FDR < 1 × 10^−5^, were included in the heatmap and the downstream enrichment analysis. Enrichment analysis for modules of KO terms that we identified was performed as described above for DEGs.

### Protein profiling using a bead-based affinity assay

Protein targets with functional associations to HIV resistance and inflammation were selected based on their presence in cervicovaginal secretions [80–83] (Suppl Table 14). Polyclonal rabbit antibodies for these proteins were generated using the Human Protein Atlas (HPA) project (The Human Protein Atlas available online: htpps://www.proteinatlas.org). For cervicovaginal lavage samples, we followed a published procedure for protein profiling of cervicovaginal secretions [80]. Antibodies were coupled to magnetic beads (MagPlex-C, Luminex Corp., Austin, TX) using EDC-NHS chemistry to create a bead array [84]. Samples were distributed in 96-well microtiter plates based on age of the participants and study group. Proteins were labeled with biotin, diluted, and heated. The bead array and the heat-treated samples were combined in a 384-well microtiter plate and incubated overnight at room temperature. A streptavidin-conjugated fluorophore (Streptavidine R-Phycoerythrin Conjugate, Invitrogen, Stockholm, Sweden) was added for the detection of the captured proteins. Bead-bound proteins were measured as fluorescence intensity (arbitrary unit) per sample and bead identity using the FlexMap 3D (Luminex Corp., Austin, USA). Data processing was performed using the open software R (version 3.6 (R: The R Project for Statistical Computing Available online: htpps://www.r-project.org/ (accessed on Nov. 20, 2020). The data was log10-transformed and normalized to reduce the differences between labeling plates as previously described [85]. Robust linear regression was applied for sample plate position (rlm function, R package MASS [86]) to minimize the effect of time delays during read-out. Possible differences in protein levels in the samples between the groups were evaluated using linear models fitted separately for each protein with the Limma package version 3.42 [87]. Women using DMPA contraception were included as a blocking variable in the model. Differentially expressed proteins were considered significant at adj. *p*-value < 0.05. Proteins with *p*-values < 0.05 were visualized in heatmaps scaled by row using base R functions.

### Cytokine measurement

Levels of a panel of 16 pre-selected cytokines (IFN**γ**, IL-12-p70, sCD40L, IL-17A, IL-1α, IL-1β, IL-2, IL-8, IP10, MCP1, MIP1α, MIP1β, TNFα, IL-1RA, MIG, MIP3α) in the cervicovaginal lavage samples were assayed using a Milliplex MAP kit (Millipore, Billerica, MA, USA) and analyzed on a BioPlex-200 (Bio-Rad, Mississauga, ON, Canada) with an overnight incubation [88]. Cytokine expression (pg/mL) showed a skewed distribution and was therefore log_2_-transformed for further analysis (zeros were replaced with 0.01 before log normalization, values below limit of detection (LOD) were replaced with value for LOD/2). A Kruskal-Wallis’ test with Benjamini Hochberg correction was used followed by a Dunn test with Benjamini Hochberg correction. Adjusted *p*-values < 0.05 were considered significant. Tests were performed in R using packages tidyverse [89] and rstatix [90].

## Supplementary Information

**Additional file 1: Supplementary Figure 1.** Abundance distribution of individual taxa in the luminal and tissue microbiome data sets. Violin plots showing the distribution of relative abundance of the top 30 most abundant taxa in the luminal and tissue-adherent data sets. **Supplementary Figure 2.** Differential bacterial abundance across the luminal and tissue microbiome datasets. Differential bacterial abundance was compared between the luminal and tissue-adherent microbiome data sets. The results are shown as a) dot plots, and b) bar plots, respectively. Bacteria with log2FC above 0.25 and *p*-value < 0.01 (from the Wilcoxon’s test) were considered significantly different and were sorted by the highest expression. The color scale indicates the difference in total abundance between the datasets as a proportion, where “max” is the highest abundance of the two datasets, and the other becomes a proportion of this value. The size of the dots indicates the average abundance of the given bacteria in the given data set. **Supplementary Figure 3.** Summary of pairwise comparisons between the study groups for differentially expressed genes, GO and KEGG pathways as well as PPI analysis. The results are shown as: a) Summary of pairwise comparison between the luminal study groups, and for the b) tissue-based study groups. For both a) and b): The number of differentially expressed genes (DEGs) (p<0.01) are displayed in the hexagon shape, these were further used for GO (round shape) and KEGG pathways (number outside round shape) analysis (FDR<0.05), as well as for PPI analysis (square shape) (FDR<0.05). The luminal group in the middle circle represents “Group A” and the luminal group at the end of the line “Group B”, and the comparison represents Group A vs. Group B, i.e. Group A has X number of upregulated DEGs compared to Group B. **Supplementary Figure 4.** Functional associations of the luminal microbiome with host tissue gene expression profiles. Bacterial abundances in the luminal samples were correlated with gene expression of the top 5,000 highly variable genes from the RNAseq dataset. This generated a correlation matrix between bacteria and genes. For each bacteria, genes were ranked based on their correlation to that bacteria, followed by gene set enrichment anlaysis (GSEA) using the KEGG gene annotation database. The resulting matrix display associations between individual bacterial taxa and corresponding KEGG term as defined in the host tissue sample. The heatmap shows the normalized enrichment score (NES). Only enrichments with *p*-value < 0.05 are shown. Bacterium and pathways with less than 10 significant NES scores were omitted from the heatmap. Bacteria are grouped according to anatomical/functional activity and marked with different colors per category. **Supplementary Figure 5.** Functional associations of the tissue microbiome with host tissue gene expression profiles. Bacterial abundances in the tissue samples were correlated with the gene expression of the top 5,000 highly variable genes from the RNAseq dataset. This generated a correlation matrix between bacteria and genes. For each bacteria, genes were ranked based on their correlation to that bacteria, followed by gene set enrichment anlaysis (GSEA) using the KEGG gene annotation database. The resulting matrix display associations between individual bacterial taxa and corresponding KEGG term as defined in the host tissue sample. The heatmap shows the normalized enrichment score (NES). Only enrichments with *p*-value < 0.05 are shown. Bacterium and pathways with less than 10 significant NES scores were omitted from the heatmap. Bacteria are grouped according to anatomical/functional activity and marked with different colors according to category. **Supplementary Figure 6.** Rarefaction curves for the microbiome 16S rRNA V4 sequencing. The rarefaction curves show numbers of unique ASVs detected in each sample when simulating increasing sequencing depth. Although low abundant taxa can be undetected at low sequencing depth, they can be detected at a higher sequencing depth (x-axis). When the curve flattens out, all taxa in the sample are considered detected. a) Luminal microbiome dataset, and b) Tissue-adherent microbiome dataset. The sequencing depth was > 40,000 reads in all but nine samples for the luminal dataset, while 16 samples had fewer than 2,500 reads in the tissue-adherent microbiome dataset.

**Additional file 2: Supplementary Table 1.** Total relative abundance, alpha diversity,definition of bacterial communities in the samples, positive controls and ASV count tables luminal and tissue. **Supplementary Table 2**. Sociodemographic and clinical characteristics per study participant. **Supplementary Table 3**. Metabolic profile of the luminal microbiome. **Supplementary Table 4.** Sociodemographic and clinical characteristics of study participants included in the transcriptomic profiling at time of tissue sample collection, grouped based on their tissue microbiome. **Supplementary Table 5**. Differentially expressed genes between the luminal study groups. **Supplementary Table 6**. Pathway enrichment analysis for the differentially expressed genes across the luminal samples. **Supplementary Table 7**. Pathway enrichment analysis for the differentially expressed genes by pairwise comparisons between the luminal samples. **Supplementary Table 8**. Transcription factor protein-protein interaction (TF-PPI) network analysis by pairwise comparisons between the luminal samples. **Supplementary Table 9**. Differentially expressed genes between the tissue study groups. **Supplementary Table 10**. Pathway enrichment analysis for the differentially expressed genes across the tissue samples. **Supplementary Table 11**. Pathway enrichment analysis for the differentially expressed genes by pairwise comparisons between the tissue samples. **Supplementary Table 12**. Transcription factor protein-protein interaction (TF-PPI) network analysis by pairwise comparisons between the tissue samples. **Supplementary Table 13**. Differentially expressed genes and pathway enrichment analysis for the comparison of sample groups defined as L2T2 and L2T3. **Supplementary Table 14**. Characterization of proteins and antibodies included in the protein profiling assay, cytokine data and cytokine results. **Supplementary Table 15.** Sociodemographic and clinical characteristics of study participants included in the protein profiling at time of luminal sample collection. **Supplementary Table 16.** Comparisons of protein levels between the luminal study groups. **Supplementary Table 17**. Comparisons of protein levels between the tissue study groups.

## Data Availability

The raw microbiome sequencing data for this study has been deposited in the European Nucleotide Archive (ENA) at EMBL-EBI under accession number PRJEB50325. The raw count with taxonomic annotation is available in Suppl. Table 1. The processed transcriptomics sequencing data files can be accessed in the Gene Expression Omnibus public repository, SuperSeries ID GSE217237. Sociodemographic and clinical characteristics included in Table 1, Suppl. Table 4, Suppl. Table 15 are listed per sample in Suppl. Table 2. Cytokine values are included in Suppl. Table 14 and normalized protein values are previously published in Bradley et al. (2022), PLoS Pathog., and available for download here 10.1371/journal.ppat.1010494.s017. The raw transcriptomic sequencing data cannot be held in a public repository due to the sensitive nature of such personal data. Request for data access can be made to the Karolinska Institutet Research Data Office (contact via rdo@ki.se) and access will be granted if the request meets the requirements of the data policy. Code availability: All scripts used for the analysis can be found at https://github.com/NBISweden/tissue-adherent-VMB.
