# Supplementary figures and images for: Distinct cervical tissue-adherent and luminal microbiome communities correlate with mucosal host gene expression and protein levels in Kenyan sex workers
Source: Microbiome. 2023 Mar 31;11:67. doi: 10.1186/s40168-023-01502-4 (PMC10064689; doi:10.1186/s40168-023-01502-4)

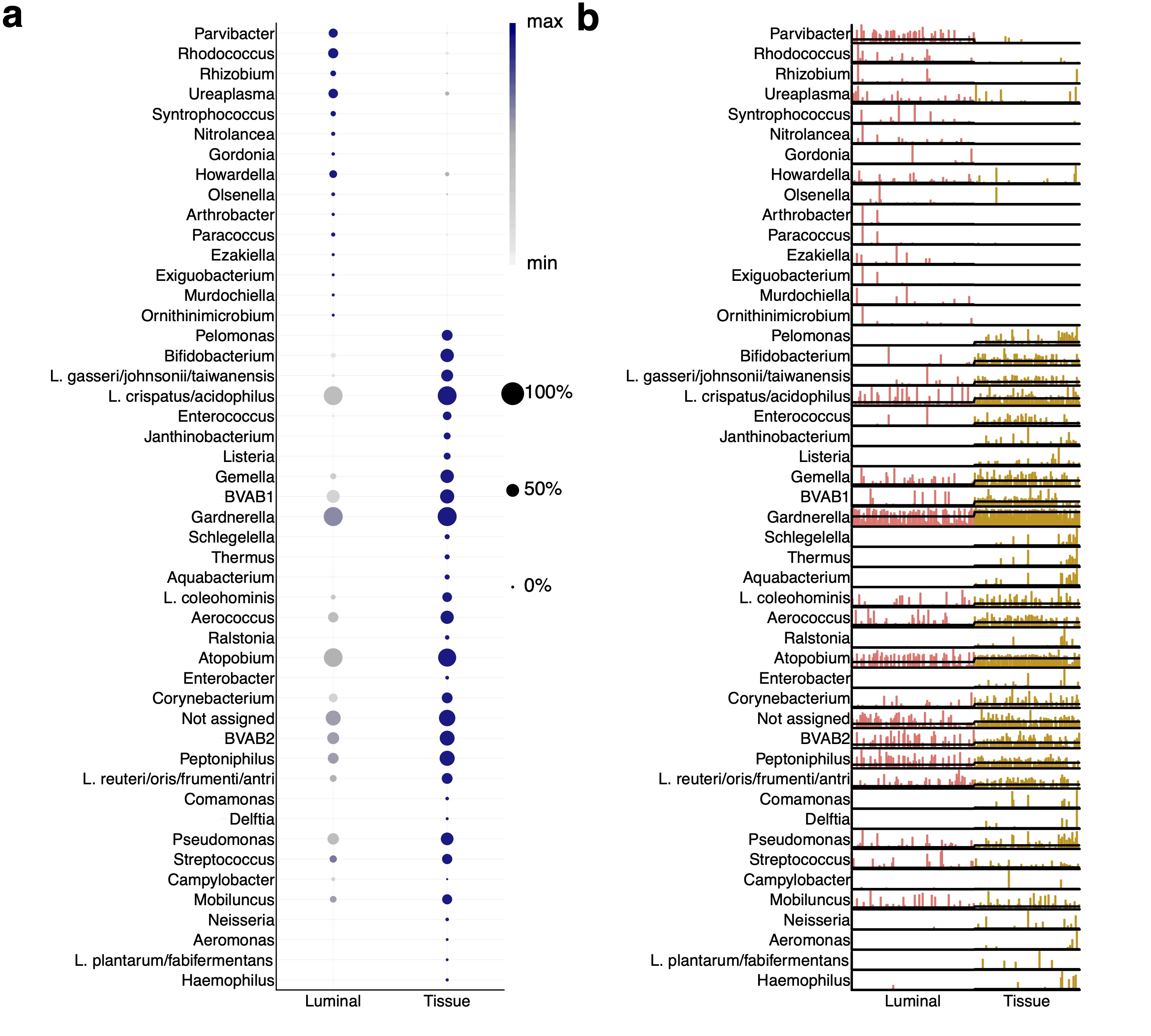

Supplement: Supplementary file 2 — Additional file 1: Supplementary Figure 1. Abundance distribution of individual taxa in the luminal and tissue microbiome data sets. Violin plots showing the distribution of relative abundance of the top 30 most abundant taxa in the luminal and tissue-adherent data sets. Supplementary Figure 2. Differential bacterial abundance across the luminal and tissue microbiome datasets. Differential bacterial abundance was compared between the luminal and tissue-adherent microbiome data sets. The results are shown as a) dot plots, and b) bar plots, respectively. Bacteria with log2FC above 0.25 and p-value < 0.01 (from the Wilcoxon’s test) were considered significantly different and were sorted by the highest expression. The color scale indicates the difference in total abundance between the datasets as a proportion, where “max” is the highest abundance of the two datasets, and the other becomes a proportion of this value. The size of the dots indicates the average abundance of the given bacteria in the given data set. Supplementary Figure 3. Summary of pairwise comparisons between the study groups for differentially expressed genes, GO and KEGG pathways as well as PPI analysis. The results are shown as: a) Summary of pairwise comparison between the luminal study groups, and for the b) tissue-based study groups. For both a) and b): The number of differentially expressed genes (DEGs) (p<0.01) are displayed in the hexagon shape, these were further used for GO (round shape) and KEGG pathways (number outside round shape) analysis (FDR<0.05), as well as for PPI analysis (square shape) (FDR<0.05). The luminal group in the middle circle represents “Group A” and the luminal group at the end of the line “Group B”, and the comparison represents Group A vs. Group B, i.e. Group A has X number of upregulated DEGs compared to Group B. Supplementary Figure 4. Functional associations of the luminal microbiome with host tissue gene expression profiles. Bacterial abundances in the luminal sampl [file 40168_2023_1502_MOESM1_ESM.zip › Suppl.Fig.2.jpg]

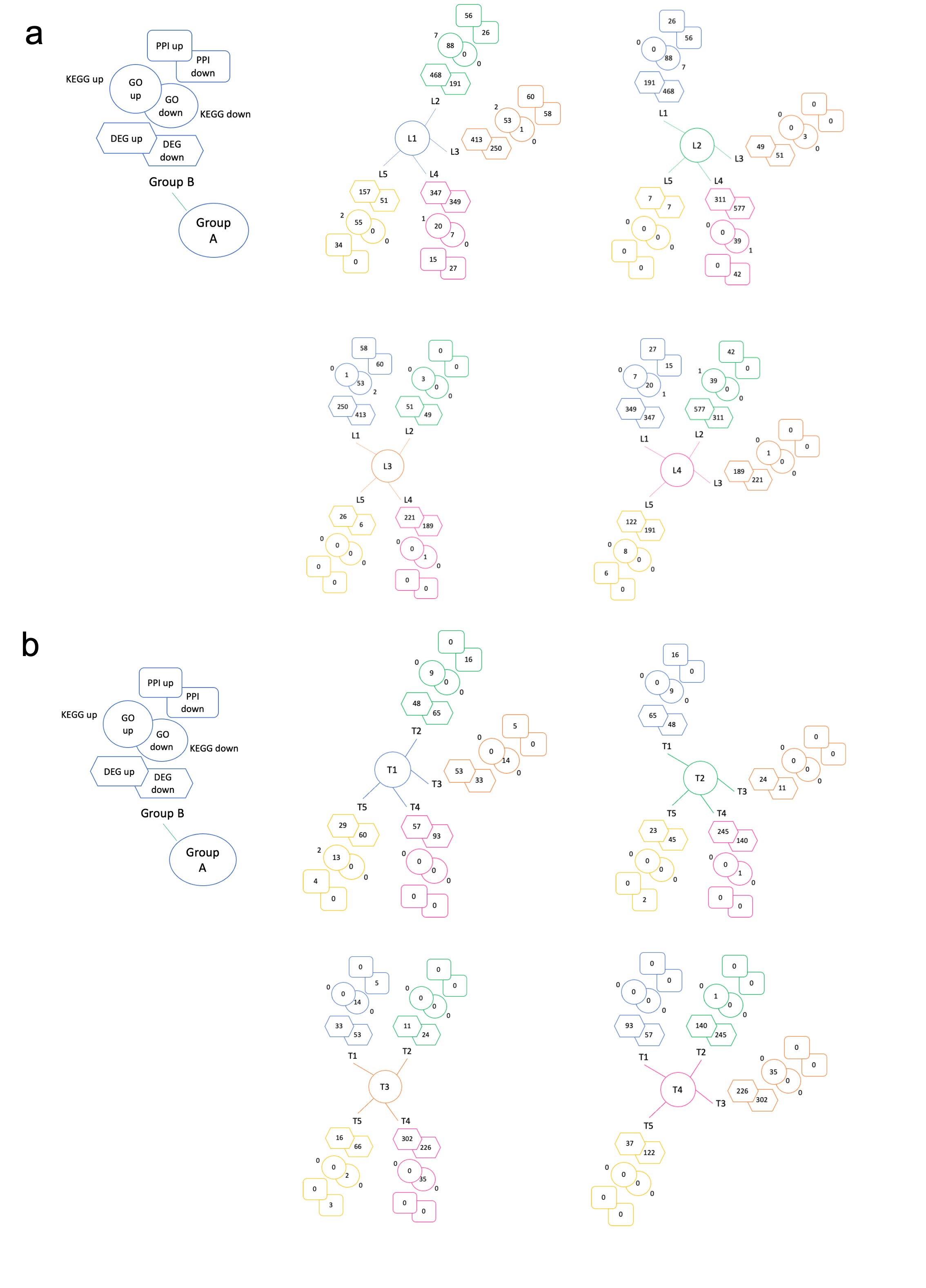

Supplement: Supplementary file 2 — Additional file 1: Supplementary Figure 1. Abundance distribution of individual taxa in the luminal and tissue microbiome data sets. Violin plots showing the distribution of relative abundance of the top 30 most abundant taxa in the luminal and tissue-adherent data sets. Supplementary Figure 2. Differential bacterial abundance across the luminal and tissue microbiome datasets. Differential bacterial abundance was compared between the luminal and tissue-adherent microbiome data sets. The results are shown as a) dot plots, and b) bar plots, respectively. Bacteria with log2FC above 0.25 and p-value < 0.01 (from the Wilcoxon’s test) were considered significantly different and were sorted by the highest expression. The color scale indicates the difference in total abundance between the datasets as a proportion, where “max” is the highest abundance of the two datasets, and the other becomes a proportion of this value. The size of the dots indicates the average abundance of the given bacteria in the given data set. Supplementary Figure 3. Summary of pairwise comparisons between the study groups for differentially expressed genes, GO and KEGG pathways as well as PPI analysis. The results are shown as: a) Summary of pairwise comparison between the luminal study groups, and for the b) tissue-based study groups. For both a) and b): The number of differentially expressed genes (DEGs) (p<0.01) are displayed in the hexagon shape, these were further used for GO (round shape) and KEGG pathways (number outside round shape) analysis (FDR<0.05), as well as for PPI analysis (square shape) (FDR<0.05). The luminal group in the middle circle represents “Group A” and the luminal group at the end of the line “Group B”, and the comparison represents Group A vs. Group B, i.e. Group A has X number of upregulated DEGs compared to Group B. Supplementary Figure 4. Functional associations of the luminal microbiome with host tissue gene expression profiles. Bacterial abundances in the luminal sampl [file 40168_2023_1502_MOESM1_ESM.zip › Suppl.Fig.3.jpg]

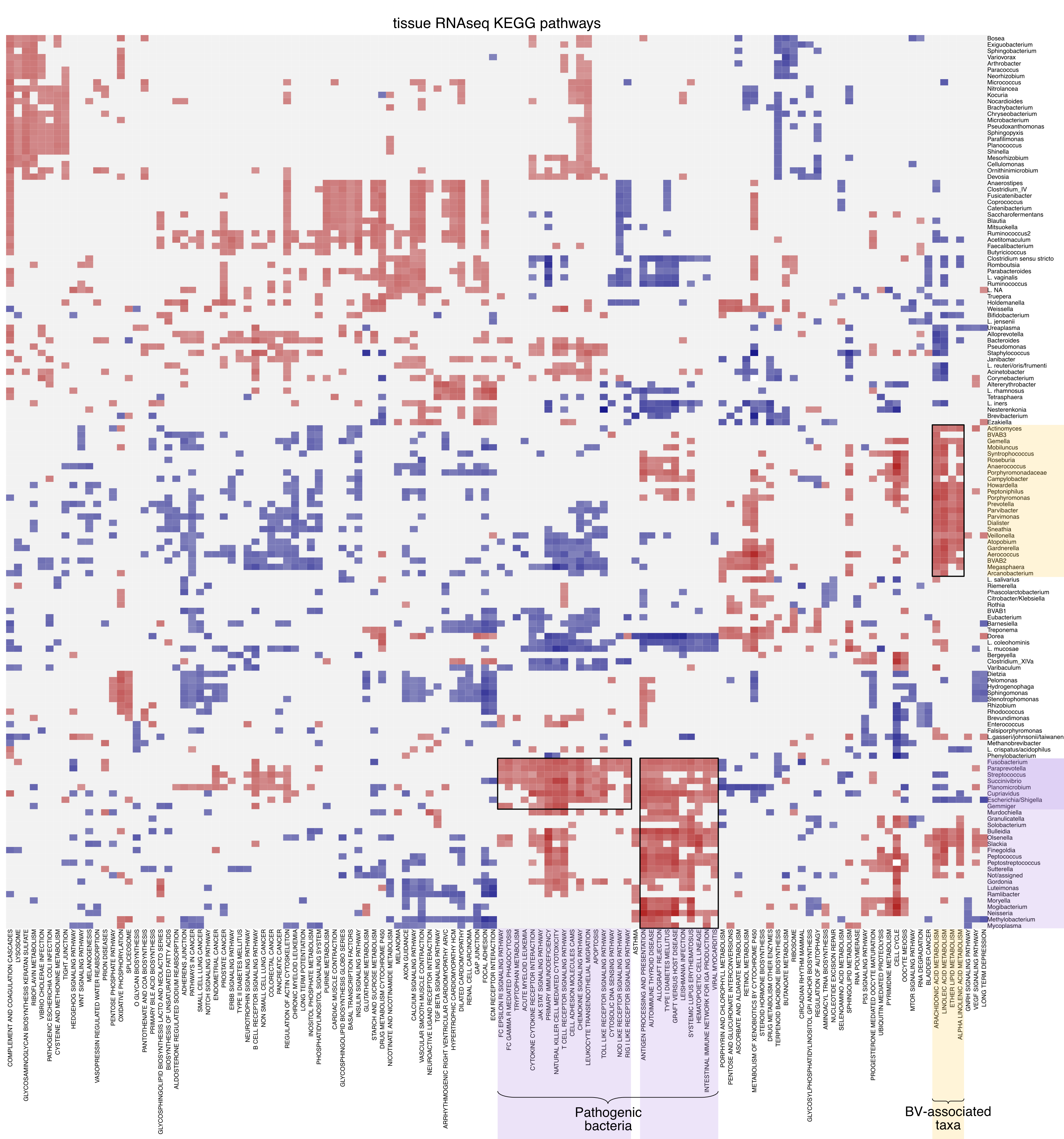

Supplement: Supplementary file 2 — Additional file 1: Supplementary Figure 1. Abundance distribution of individual taxa in the luminal and tissue microbiome data sets. Violin plots showing the distribution of relative abundance of the top 30 most abundant taxa in the luminal and tissue-adherent data sets. Supplementary Figure 2. Differential bacterial abundance across the luminal and tissue microbiome datasets. Differential bacterial abundance was compared between the luminal and tissue-adherent microbiome data sets. The results are shown as a) dot plots, and b) bar plots, respectively. Bacteria with log2FC above 0.25 and p-value < 0.01 (from the Wilcoxon’s test) were considered significantly different and were sorted by the highest expression. The color scale indicates the difference in total abundance between the datasets as a proportion, where “max” is the highest abundance of the two datasets, and the other becomes a proportion of this value. The size of the dots indicates the average abundance of the given bacteria in the given data set. Supplementary Figure 3. Summary of pairwise comparisons between the study groups for differentially expressed genes, GO and KEGG pathways as well as PPI analysis. The results are shown as: a) Summary of pairwise comparison between the luminal study groups, and for the b) tissue-based study groups. For both a) and b): The number of differentially expressed genes (DEGs) (p<0.01) are displayed in the hexagon shape, these were further used for GO (round shape) and KEGG pathways (number outside round shape) analysis (FDR<0.05), as well as for PPI analysis (square shape) (FDR<0.05). The luminal group in the middle circle represents “Group A” and the luminal group at the end of the line “Group B”, and the comparison represents Group A vs. Group B, i.e. Group A has X number of upregulated DEGs compared to Group B. Supplementary Figure 4. Functional associations of the luminal microbiome with host tissue gene expression profiles. Bacterial abundances in the luminal sampl [file 40168_2023_1502_MOESM1_ESM.zip › Suppl.Fig.4.jpg]

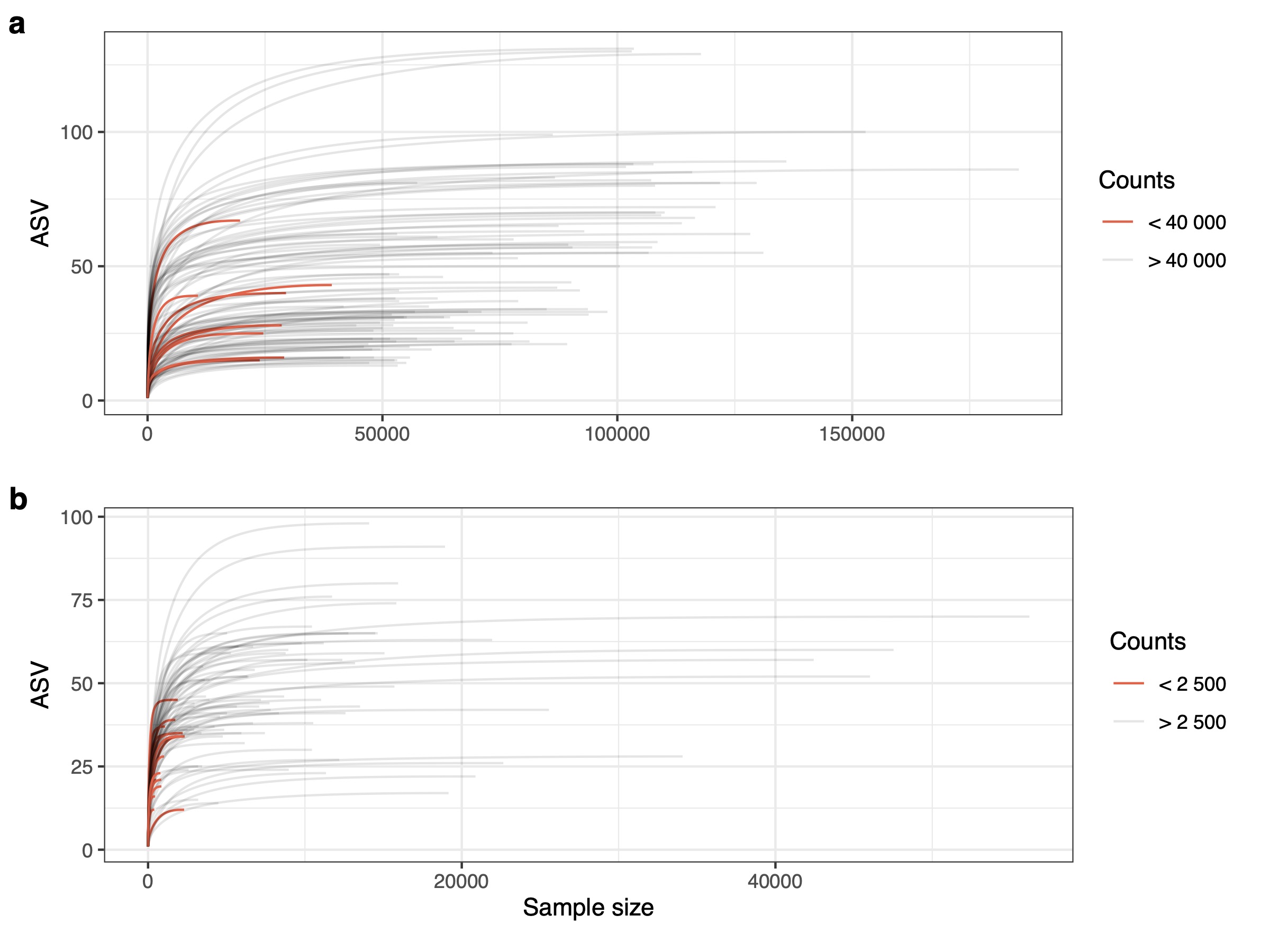

Supplement: Supplementary file 2 — Additional file 1: Supplementary Figure 1. Abundance distribution of individual taxa in the luminal and tissue microbiome data sets. Violin plots showing the distribution of relative abundance of the top 30 most abundant taxa in the luminal and tissue-adherent data sets. Supplementary Figure 2. Differential bacterial abundance across the luminal and tissue microbiome datasets. Differential bacterial abundance was compared between the luminal and tissue-adherent microbiome data sets. The results are shown as a) dot plots, and b) bar plots, respectively. Bacteria with log2FC above 0.25 and p-value < 0.01 (from the Wilcoxon’s test) were considered significantly different and were sorted by the highest expression. The color scale indicates the difference in total abundance between the datasets as a proportion, where “max” is the highest abundance of the two datasets, and the other becomes a proportion of this value. The size of the dots indicates the average abundance of the given bacteria in the given data set. Supplementary Figure 3. Summary of pairwise comparisons between the study groups for differentially expressed genes, GO and KEGG pathways as well as PPI analysis. The results are shown as: a) Summary of pairwise comparison between the luminal study groups, and for the b) tissue-based study groups. For both a) and b): The number of differentially expressed genes (DEGs) (p<0.01) are displayed in the hexagon shape, these were further used for GO (round shape) and KEGG pathways (number outside round shape) analysis (FDR<0.05), as well as for PPI analysis (square shape) (FDR<0.05). The luminal group in the middle circle represents “Group A” and the luminal group at the end of the line “Group B”, and the comparison represents Group A vs. Group B, i.e. Group A has X number of upregulated DEGs compared to Group B. Supplementary Figure 4. Functional associations of the luminal microbiome with host tissue gene expression profiles. Bacterial abundances in the luminal sampl [file 40168_2023_1502_MOESM1_ESM.zip › Suppl.Fig.6.jpg]

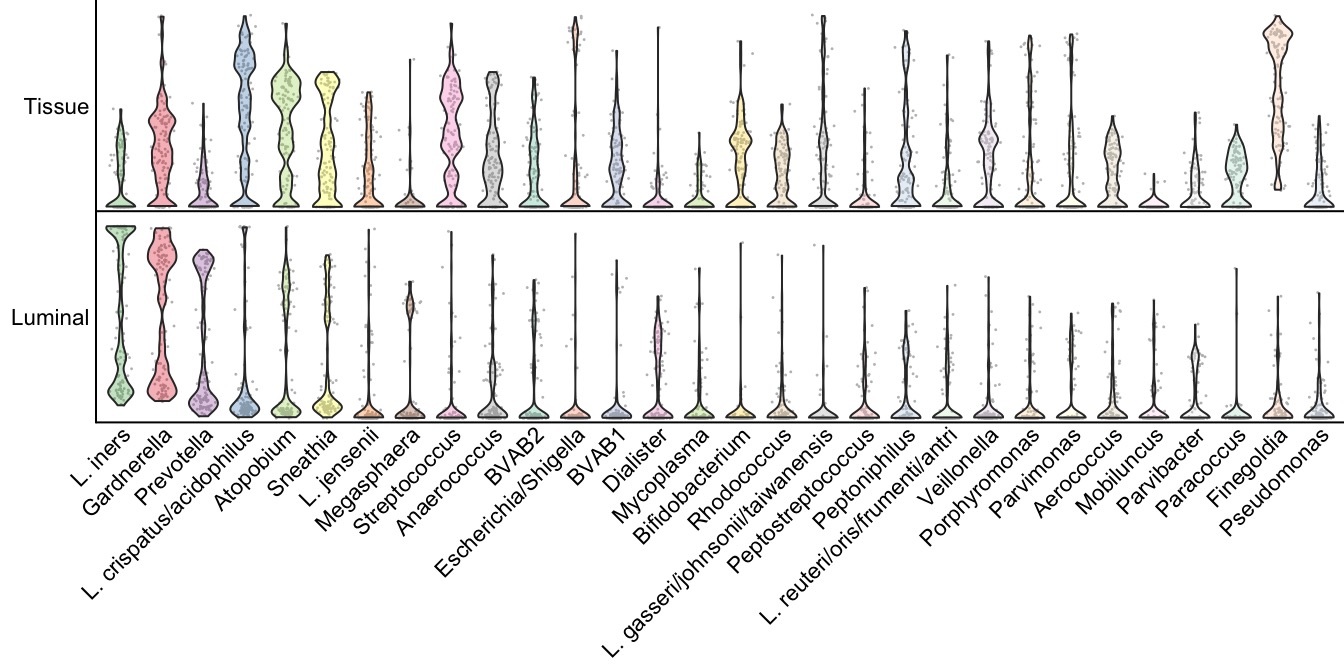

Supplement: Supplementary file 2 — Additional file 1: Supplementary Figure 1. Abundance distribution of individual taxa in the luminal and tissue microbiome data sets. Violin plots showing the distribution of relative abundance of the top 30 most abundant taxa in the luminal and tissue-adherent data sets. Supplementary Figure 2. Differential bacterial abundance across the luminal and tissue microbiome datasets. Differential bacterial abundance was compared between the luminal and tissue-adherent microbiome data sets. The results are shown as a) dot plots, and b) bar plots, respectively. Bacteria with log2FC above 0.25 and p-value < 0.01 (from the Wilcoxon’s test) were considered significantly different and were sorted by the highest expression. The color scale indicates the difference in total abundance between the datasets as a proportion, where “max” is the highest abundance of the two datasets, and the other becomes a proportion of this value. The size of the dots indicates the average abundance of the given bacteria in the given data set. Supplementary Figure 3. Summary of pairwise comparisons between the study groups for differentially expressed genes, GO and KEGG pathways as well as PPI analysis. The results are shown as: a) Summary of pairwise comparison between the luminal study groups, and for the b) tissue-based study groups. For both a) and b): The number of differentially expressed genes (DEGs) (p<0.01) are displayed in the hexagon shape, these were further used for GO (round shape) and KEGG pathways (number outside round shape) analysis (FDR<0.05), as well as for PPI analysis (square shape) (FDR<0.05). The luminal group in the middle circle represents “Group A” and the luminal group at the end of the line “Group B”, and the comparison represents Group A vs. Group B, i.e. Group A has X number of upregulated DEGs compared to Group B. Supplementary Figure 4. Functional associations of the luminal microbiome with host tissue gene expression profiles. Bacterial abundances in the luminal sampl [file 40168_2023_1502_MOESM1_ESM.zip › Suppl.Fig.1.jpeg]

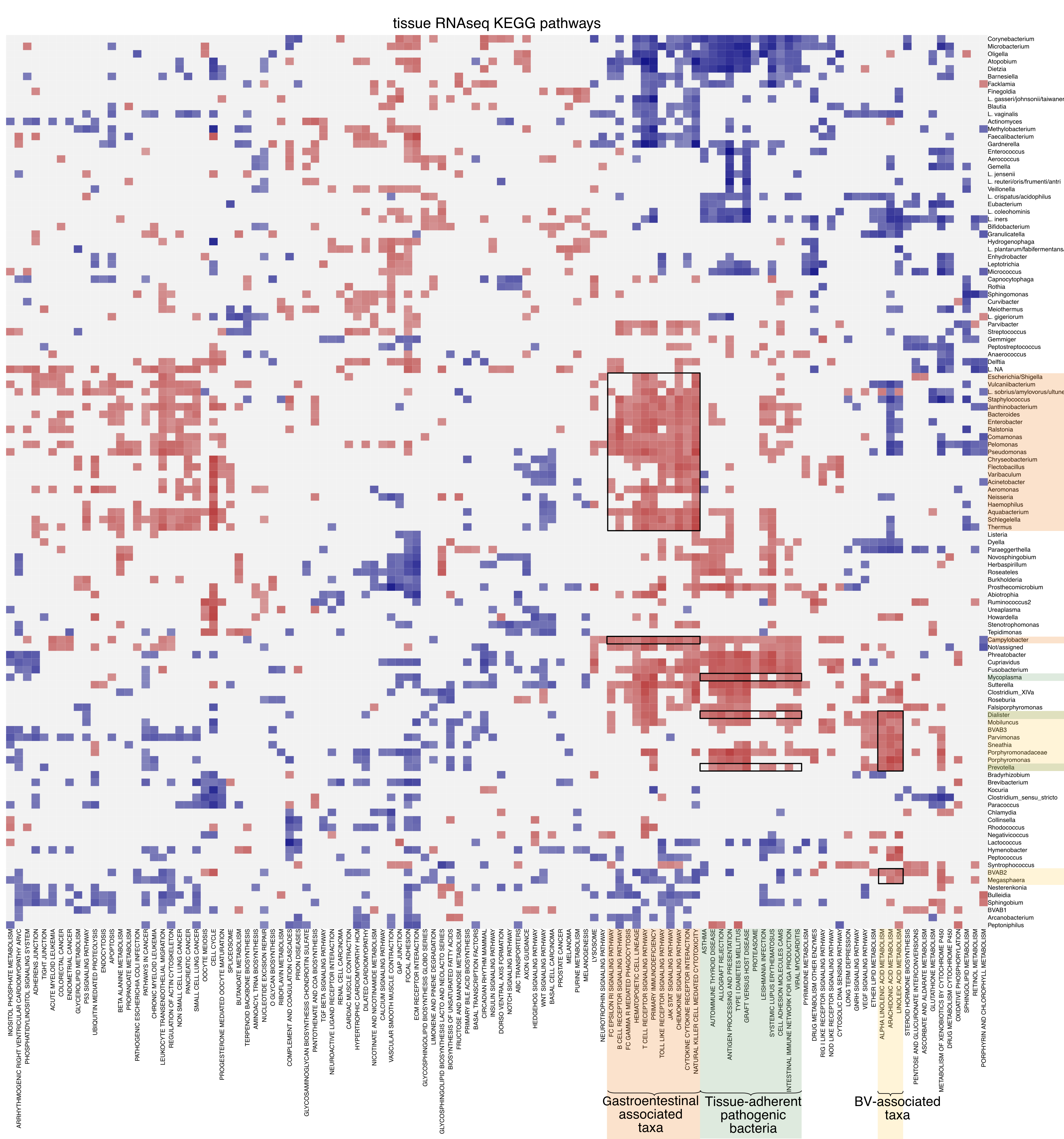

Supplement: Supplementary file 2 — Additional file 1: Supplementary Figure 1. Abundance distribution of individual taxa in the luminal and tissue microbiome data sets. Violin plots showing the distribution of relative abundance of the top 30 most abundant taxa in the luminal and tissue-adherent data sets. Supplementary Figure 2. Differential bacterial abundance across the luminal and tissue microbiome datasets. Differential bacterial abundance was compared between the luminal and tissue-adherent microbiome data sets. The results are shown as a) dot plots, and b) bar plots, respectively. Bacteria with log2FC above 0.25 and p-value < 0.01 (from the Wilcoxon’s test) were considered significantly different and were sorted by the highest expression. The color scale indicates the difference in total abundance between the datasets as a proportion, where “max” is the highest abundance of the two datasets, and the other becomes a proportion of this value. The size of the dots indicates the average abundance of the given bacteria in the given data set. Supplementary Figure 3. Summary of pairwise comparisons between the study groups for differentially expressed genes, GO and KEGG pathways as well as PPI analysis. The results are shown as: a) Summary of pairwise comparison between the luminal study groups, and for the b) tissue-based study groups. For both a) and b): The number of differentially expressed genes (DEGs) (p<0.01) are displayed in the hexagon shape, these were further used for GO (round shape) and KEGG pathways (number outside round shape) analysis (FDR<0.05), as well as for PPI analysis (square shape) (FDR<0.05). The luminal group in the middle circle represents “Group A” and the luminal group at the end of the line “Group B”, and the comparison represents Group A vs. Group B, i.e. Group A has X number of upregulated DEGs compared to Group B. Supplementary Figure 4. Functional associations of the luminal microbiome with host tissue gene expression profiles. Bacterial abundances in the luminal sampl [file 40168_2023_1502_MOESM1_ESM.zip › Suppl.Fig.5.jpg]
