# Supplementary material for: Distinct cervical tissue-adherent and luminal microbiome communities correlate with mucosal host gene expression and protein levels in Kenyan sex workers
Source: Microbiome. 2023 Mar 31;11:67. doi: 10.1186/s40168-023-01502-4 (PMC10064689; doi:10.1186/s40168-023-01502-4)
Supplement: Supplementary file 3 — Additional file 2: Supplementary Table 1. Total relative abundance, alpha diversity,definition of bacterial communities in the samples, positive controls and ASV count tables luminal and tissue. Supplementary Table 2. Sociodemographic and clinical characteristics per study participant. Supplementary Table 3. Metabolic profile of the luminal microbiome. Supplementary Table 4. Sociodemographic and clinical characteristics of study participants included in the transcriptomic profiling at time of tissue sample collection, grouped based on their tissue microbiome. Supplementary Table 5. Differentially expressed genes between the luminal study groups. Supplementary Table 6. Pathway enrichment analysis for the differentially expressed genes across the luminal samples. Supplementary Table 7. Pathway enrichment analysis for the differentially expressed genes by pairwise comparisons between the luminal samples. Supplementary Table 8. Transcription factor protein-protein interaction (TF-PPI) network analysis by pairwise comparisons between the luminal samples. Supplementary Table 9. Differentially expressed genes between the tissue study groups. Supplementary Table 10. Pathway enrichment analysis for the differentially expressed genes across the tissue samples. Supplementary Table 11. Pathway enrichment analysis for the differentially expressed genes by pairwise comparisons between the tissue samples. Supplementary Table 12. Transcription factor protein-protein interaction (TF-PPI) network analysis by pairwise comparisons between the tissue samples. Supplementary Table 13. Differentially expressed genes and pathway enrichment analysis for the comparison of sample groups defined as L2T2 and L2T3. Supplementary Table 14. Characterization of proteins and antibodies included in the protein profiling assay, cytokine data and cytokine results. Supplementary Table 15. Sociodemographic and clinical characteristics of study participants included in the protein profiling at time of lumi [file 40168_2023_1502_MOESM2_ESM.zip › Suppl tbls/Suppl.Tbl.15 Clin tbl lumin prot.docx]

**Supplementary Table 14. Sociodemographic and clinical characteristics of study participants at time of luminal sample collection for protein analysis**

**Study groups**

**L1 L2 L3 L4 L5 p-value**^a^ **Data n/a**^b^

(n=8) (n=26) (n=18) (n=27) (n=5)

median (range or %) number

***Sociodemographic parameters***

**Age** (years) 31 (23-47) 30 (21-45) 34 (20-48) 32 (21-50) 29 (24-40) 0.31^1^ 0

**Time in sex work** (months) 24 (12-36) 24 (8-264) 96 (12-372) 24 (3-324) 24 (12-36) 0.16^1^ 1

**Number of weekly clients**^c^ 9 (3-25) 4 (1-30) 5 (0-30) 4 (0-14) 3 (2-28) 0.35^1^ 7

**Marital status** (married) 1 (12%) 4 (15%) 3 (17%) 6 (22%) 2 (40%) 0.72^2^ 2

**Children** (number) 2 (0-3) 1 (1-3) 2 (1-4) 2 (0-4) 2 (1-5) 0.66^1^ 13

**Educational level** (years in school) 10 (8-16) 10 (7-15) 9 (7-15) 11 (7-21) 11 (6-15) 0.63^1^ 1

***Sex hormone status***

**DMPA use** (yes) 3 (38%) 11 (42%) 6 (33%) 8 (30%) 4 (80%) 0.3^2^ 0

**Progesterone**^d^ (ng/mL) 0.05(0.05-0.05) 0.05(0.05-0.05) 0.05(0.05-0.09) 0.05(0.05-0.05) 0.05(0.05-0.05) 0.38^1^ 1

**Estradiol**^d^ (pg/mL) 22 (22-68) 22 (22-124) 29 (22-35) 22 (22-92) 31 (22-57) 0.96^1^ 1

**Time since onset of menses**^e^ (days) 16 (9-44) 7 (5-40) 11 (5-30) 8 (4-34) 6 (6-6) 0.08^1^ 4

**Progesteron**^f^ (ng/mL) 3 (0.05-8) 0.05(0.05-9) 0.05(0.05-19) 0.05(0.05-11) 0.15(0.15-0.15) 0.24^1^ 0

**Estradiol**^f^ (pg/mL) 209(36-224) 73(22-258) 140(22-405) 93(22-248) 248(248-248) 0.24^1^ 0

***STIs and vaginal health***

**HIV serostatus** (seropositive) 0 0 0 0 0 0

**Presence of NG**^g^ 0 0 0 0 0 0

**Presence of CT**^g^ 0 0 0 0 0 0

**Presence of yeast**^g^ 0 0 0 1 (4%) 1 (20%) 0.04^2^ 2

**Vaginal discharge**^g^ 2 (25%) 1 (4%) 1 (6%) 2 (7%) 0 0.37^2^ 5

**Nugents’ score (bacterial vaginosis)**^h^ <0.001^2^ 2

-negative (0-3) 5 (62%) 26 (100%) 8 (44%) 0 1 (20%)

*-*intermediate (4-6) 2 (25%) 0 8 (44%) 7 (26%) 2 (40%)

*-*positive (7-10) 0 0 2 (11%) 20 (74%) 1 (20%)

^a^p-values: ^1^Kruskal-Wallis Rank Sum Test; ^2^Fischer’s exact test

^b^Data n/a: Data not available for number of samples

^c^Number of weekly clients: Data from the questionnaire two weeks prior to sample collection: ”How many clients did you have the past 7 days?”

^d^Plasma concentration of progesterone (P4, lower limit of detection=0.05 ng/mL) and estradiol (E2, lower limit of detection 22 pg/mL) in study participants using DMPA.

^e^Time since onset of last menses (days) for study particpants not using hormonal contraceptives (the control group)

^f^Plasma concentration of progesterone (P4, lower limit of detection=0.05 ng/mL) and estradiol (E2, lower limit of detection 22 pg/mL) in study participants not using hormonal contraceptives (the control group).

^g^Having an ongoing STI at time of enrolment was an exclusion criteria for participating in the study. None of the study participants were diagnosed with neither *C.trachomatis* nor *N. gonorroheae* at time of sample collection. The scoring for yeast was made on the Gram-stained slide used for BV. Presence of discharge was recorded during physical examination.

^h^Bacterial vaginosis: the statistical analysis is based on BV diagnosis (Nugent’s score 7-10) (yes/no)
